# Minimally invasive power sources for implantable electronics

**DOI:** 10.1002/EXP.20220106

**Published:** 2023-08-31

**Authors:** Ming Xu, Yuheng Liu, Kai Yang, Shaoyin Li, Manman Wang, Jianan Wang, Dong Yang, Maxim Shkunov, S. Ravi P. Silva, Fernando A. Castro, Yunlong Zhao

**Affiliations:** ^1^ Advanced Technology Institute University of Surrey Guildford Surrey UK; ^2^ National Physical Laboratory Teddington Middlesex UK; ^3^ Dyson School of Design Engineering Imperial College London London UK; ^4^ Department of Chemical and Process Engineering University of Surrey Guildford Surrey UK; ^5^ Department of Environmental Science and Engineering Xi'an Jiaotong University Xi'an China; ^6^ The Key Laboratory of Biomedical Information Engineering of Ministry of Education School of Life Science and Technology Xi'an Jiaotong University Xi'an China

**Keywords:** energy harvesting, energy storage, implantable electronics, power source, wireless power

## Abstract

As implantable medical electronics (IMEs) developed for healthcare monitoring and biomedical therapy are extensively explored and deployed clinically, the demand for non‐invasive implantable biomedical electronics is rapidly surging. Current rigid and bulky implantable microelectronic power sources are prone to immune rejection and incision, or cannot provide enough energy for long‐term use, which greatly limits the development of miniaturized implantable medical devices. Herein, a comprehensive review of the historical development of IMEs and the applicable miniaturized power sources along with their advantages and limitations is given. Despite recent advances in microfabrication techniques, biocompatible materials have facilitated the development of IMEs system toward non‐invasive, ultra‐flexible, bioresorbable, wireless and multifunctional, progress in the development of minimally invasive power sources in implantable systems has remained limited. Here three promising minimally invasive power sources summarized, including energy storage devices (biodegradable primary batteries, rechargeable batteries and supercapacitors), human body energy harvesters (nanogenerators and biofuel cells) and wireless power transfer (far‐field radiofrequency radiation, near‐field wireless power transfer, ultrasonic and photovoltaic power transfer). The energy storage and energy harvesting mechanism, configurational design, material selection, output power and in vivo applications are also discussed. It is expected to give a comprehensive understanding of the minimally invasive power sources driven IMEs system for painless health monitoring and biomedical therapy with long‐term stable functions.

## Graphical abstract

This review paper provides a comprehensive overview of the historical development of implantable medical electronics (IMEs) and three main categories of applicable alternative minimally invasive power sources. A detailed discussion of energy storage and harvesting mechanism, configurational design, output power and in vivo applications is given. An outlook based on the current advancements and limitations is also presented.

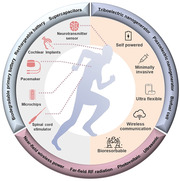

## 1 INTRODUCTION

Since Hans Berger introduced electroencephalography (EEG) to record the electrical activity of the brain in 1929, electronics for physiological measurement and biological stimulation have been of interest for more than 90 years.^[^ ^1^, ^2^, ^3^ ^]^ Over the past decades, numerous wearable and implantable electronics were designed for monitoring and measuring biological signals. According to Halperin et al., over 25 million United State citizens rely on implantable medical devices for life‐critical functions in 2008,^[^ ^4^ ^]^ the number of implantable cardioverter defibrillator implants increased ten‐times between 1990 and 2002,^[^ ^5^, ^6^ ^]^ the demand for implantable medical electronics (IMEs) in 2015 in the United State is about $52B.^[^ ^7^ ^]^ At present, the implantable medical device market is not just orientated to the growing geriatric population and associated prevalence of chronic degenerative diseases, younger populations pursuing a healthier and high‐tech lifestyle also drive the lucrative wearable and implantable electronics market. The state‐of‐art technologies enabled biological signal recording including electrophysiological, physiological (pulse, temperature), mechanical (strain, pressure) and biochemical (glucose, pH) information.^[^ ^8^, ^9^, ^10^ ^]^ Each of these biological signals is of vital importance for clinical research about physical functioning and various diseases.

However, it is still challenging to get access to bio‐signals with high fidelity and stability from the target area of living organisms while conventional IMEs still stay rigid and bulky. The recent advanced technologies in microelectromechanical systems (MEMS), ultrathin electronic devices, sensors and biocompatible/bioresorbable encapsulating layers have broadened the application range of implantable electronics from traditional rigid and bulky devices to soft bioelectronics systems that interface with the complex geometries and curved surfaces of the human body.^[^ ^8^, ^11^ ^]^ Compared with the first cardiac pacemaker invented and implanted in the human body in 1958,^[^ ^12^ ^]^ bio‐integrated electronics with good flexibility and stretchability show huge progress and more benefits for precise signal recording and pain alleviation from patients. Nowadays, numerous miniaturized implants and wearable electronics have been developed. For instance, electrocorticography (ECoG) and electrocardiogram (ECG) sensors for neurological disorders study (e.g. Epilepsy, dementia, Parkinson's disease and restless leg syndrome), angioplasty tools, prosthetic eye/skin, optoelectronic nerve stimulator, wearable pressure, strain, temperature sensors combined with drug delivery and data storage devices, signal recording and real‐time treatment integrated with wireless data transmission, and wearable energy storage system for portable and remote healthcare.^[^ ^8^, ^13^, ^14^ ^]^ Though great progress has been made over the past decades to minimize the dimension of implants, a higher requirement towards more precise biomedical functions has also been set. The dimensions of the medical electronic system probing these signals are still orders of magnitude larger than those cells or tissues, thus developing minimally invasive or injectable micro and nanoscale medical electronic systems closely matching target tissues is necessary for the evolution of new‐generation bioelectronics.

To provide energy for the new‐generation minimally invasive bioelectronics system, the size and weight of the power source as a significant part of this system should also be taken into consideration. Batteries as the most used electrochemical energy storage devices developed for IMEs have enabled the successful operation for the treatment of human disease. Though energy requirements for the power sources vary with the IMEs functions, high volumetric energy density and minimized dimension are highlighted aiming to minimize discomfort for the patient.^[^ ^15^ ^]^ However, conventional energy storage devices (such as Li‐ion batteries) are usually large and bulky with inflexible packaging, leading to associated issues such as secondary invasive surgery for replacement due to limited capacity and potentially toxic substances leakage risk.^[^ ^16^ ^]^ Therefore, new challenges have been present for power source devices to match with the soft, 3D and dynamically curved biological organisms while providing enough energy to the biomedical system.^[^ ^17^ ^]^ To overcome the key limitations to the development of a minimally invasive IMEs system, the prospective implantable power sources should be developed towards the following features: minimally invasive, lightweight, durable, high‐capacity, flexible, biocompatible or bioresorbable.

According to the different functional requirements of the IMEs, the designed and matched power source for the system varied. For short‐term implantable electronics that are designed to be implanted and operate only for a prescribed time, biodegradable and bioabsorbable materials provide a unique opportunity. For instance, applications like muscle stimulation, bone growth stimulation, neurostimulation and wound healing require the power source to operate only for a short time compared with heart stimulation working over the whole patient's life, biodegradable and bioabsorbable electronics can act as a desirable option to avoid the second surgery for device retrieving and tissue lesion.^[^ ^18^ ^]^ As physically transient electronics, biodegradable power sources including biodegradable primary batteries,^[^ ^19^, ^20^, ^21^, ^22^ ^]^ biodegradable supercapacitors^[^ ^23^ ^]^ and bioresorbable power harvesters,^[^ ^24^ ^]^ can be fully dissolved into biologically benign byproducts in biofluids through hydrolysis and thus well resolve the problem of repeated surgery without secondary invasion. For long‐term applications, durable energy storage devices with high energy density and energy harvesting devices with long‐term stability are necessary. Compared with the primary battery, the rechargeable battery can provide a longer serving time and has been developed for neurostimulators operating in the milliwatt power range.^[^ ^15^ ^]^ But for the minimally invasive implantable electronic system, secondary rechargeable batteries must also satisfy the requirements of bioabsorbable primary batteries, including reduced invasion and biocompatibility except for high energy density. In this case, miniaturized rechargeable batteries with a high aspect ratio form can well solve the problem occurred with traditional bulky lithium‐ion batteries and achieve charge/discharge cycling process in vivo such as sodium‐ion batteries and fibre supercapacitors showed decent power capability and good biocompatibility.^[^ ^25^, ^26^, ^27^ ^]^ Alternatively, the energy sources from the human body are also promising power options, which can provide a continuous stream of energy for medical devices such as pacemaker without restrictions like battery replacement and cumbersome daily charging.^[^ ^28^ ^]^ Emerging biocompatible, ultra‐flexible and miniaturized energy harvesters include piezoelectric nanogenerators (PENG) and triboelectric nanogenerators (TENG) for mechanical energy harvesting and biofuel cell for chemical energy harvesting.^[^ ^29^, ^30^, ^31^, ^32^, ^33^, ^34^ ^]^ The human body as a natural energy conversion factory can provide an abundant and constant flow of available biochemical and kinetic energy, it provides unlimited possibilities to support the IMEs during the whole lifetime of human and even to prolong the human lifespan.^[^ ^28^ ^]^ In addition, wireless and battery‐free technologies have also been widely studied for the application in miniaturized and ultralightweight devices probing signals with high chronic stability and signal fidelity.^[^ ^35^, ^36^ ^]^ For electronics such as optogenetic stimulator interfacing with neural tissues and micro injectable needles for multisite recording on the cellular scale, wireless power transmission technologies can provide reliable and constant power supply with versatile design and deployment options. At present, four main kinds of wireless power transmission technologies have been developed including near‐field magnetic resonant coupling,^[^ ^37^ ^]^ far‐field radio frequency (RF),^[^ ^38^, ^39^ ^]^ photovoltaic (PV) power transfer,^[^ ^40^ ^]^ and ultrasonic power transfer.^[^ ^41^ ^]^ It can be applied as independent energy supply but also as complementary recharging technology to extend the lifespan of implanted energy storage devices.

**FIGURE 1:**
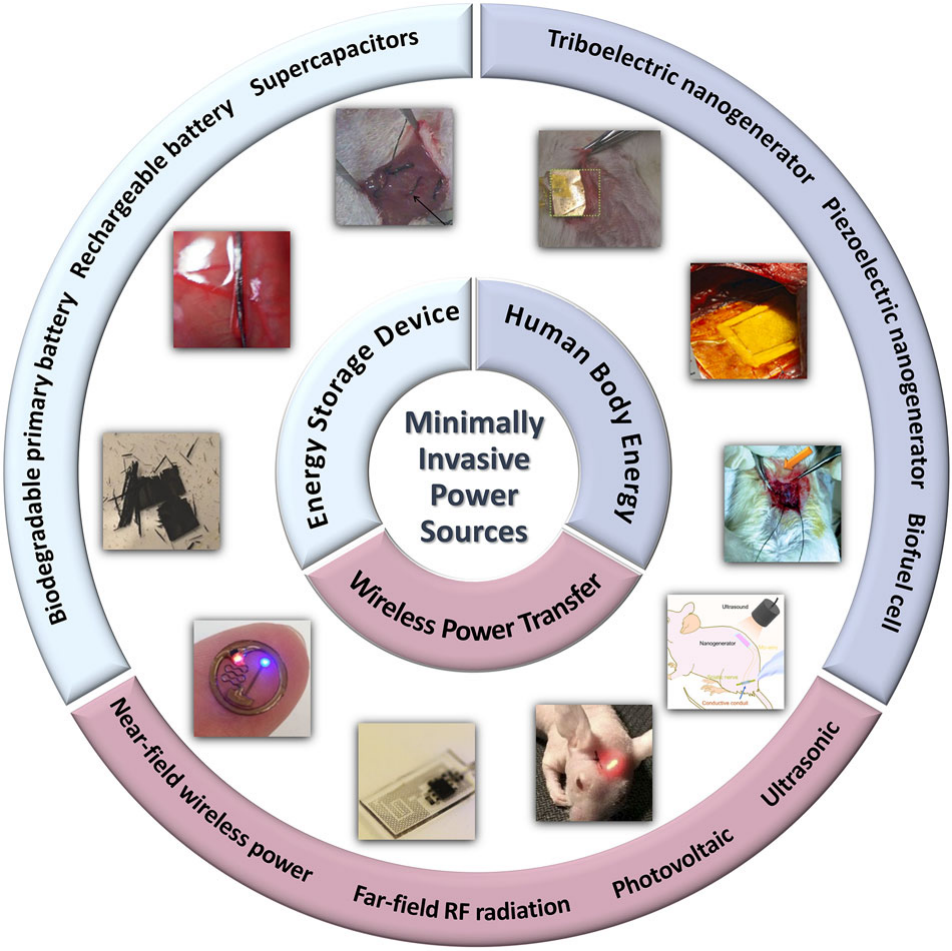
Overview of various alternative minimally invasive power sources. “Biodegradable primary battery” Reproduced with permission.^[^ ^19^ ^]^ Copyright 2014, Wiley‐VCH GmbH. “Rechargeable battery” Reproduced with permission.^[^ ^26^ ^]^ Copyright 2021, Royal Society of Chemistry. “Supercapacitors” Reproduced with permission.^[^ ^27^ ^]^ Copyright 2018, Elsevier. “Triboelectric nanogenerator” Reproduced with permission.^[^ ^30^ ^]^ Copyright 2014, Wiley‐VCH GmbH. “Piezoelectric nanogenerator” Reproduced with permission.^[^ ^29^ ^]^ Copyright 2021, Elsevier. “Biofuel cell” Reproduced with permission.^[^ ^31^ ^]^ Copyright 2013, Royal Society of Chemistry. “Ultrasonic wireless power” Reproduced with permission.^[^ ^41^ ^]^ Copyright 2022, Elsevier. “Photovoltaic wireless power” Reproduced with permission.^[^ ^40^ ^]^ Copyright 2018, Proceedings of the National Academy of Sciences. “Far‐field RF radiation wireless power” Reproduced with permission.^[^ ^39^ ^]^ Copyright 2015, Springer Nature. “Near‐field wireless power” Reproduced with permission.^[^ ^37^ ^]^ Copyright 2017, Elsevier.

In this review, we provided an overview of the state‐of‐the‐art minimally invasive implantable electronics with an outlook of the smart and injury‐reduced implantable electronics in the future, and aim to summarize various alternative miniaturized power sources for the implantable electronic system. Three broad categories of minimally invasive power sources were classified regarding their different energy supply mechanism: Energy storage devices, human body energy and wireless power transfer as summarized in Figure 1. The advantages and limitations of each kind of power strategy were reviewed in detail. Finally, the challenges and prospective future of minimally invasive electronics and corresponding applicable power sources were discussed.

## 2 DEVELOPMENT HISTORY AND RECENT PROGRESS IN IMPLANTABLE ELECTRONICS

**FIGURE 2:**
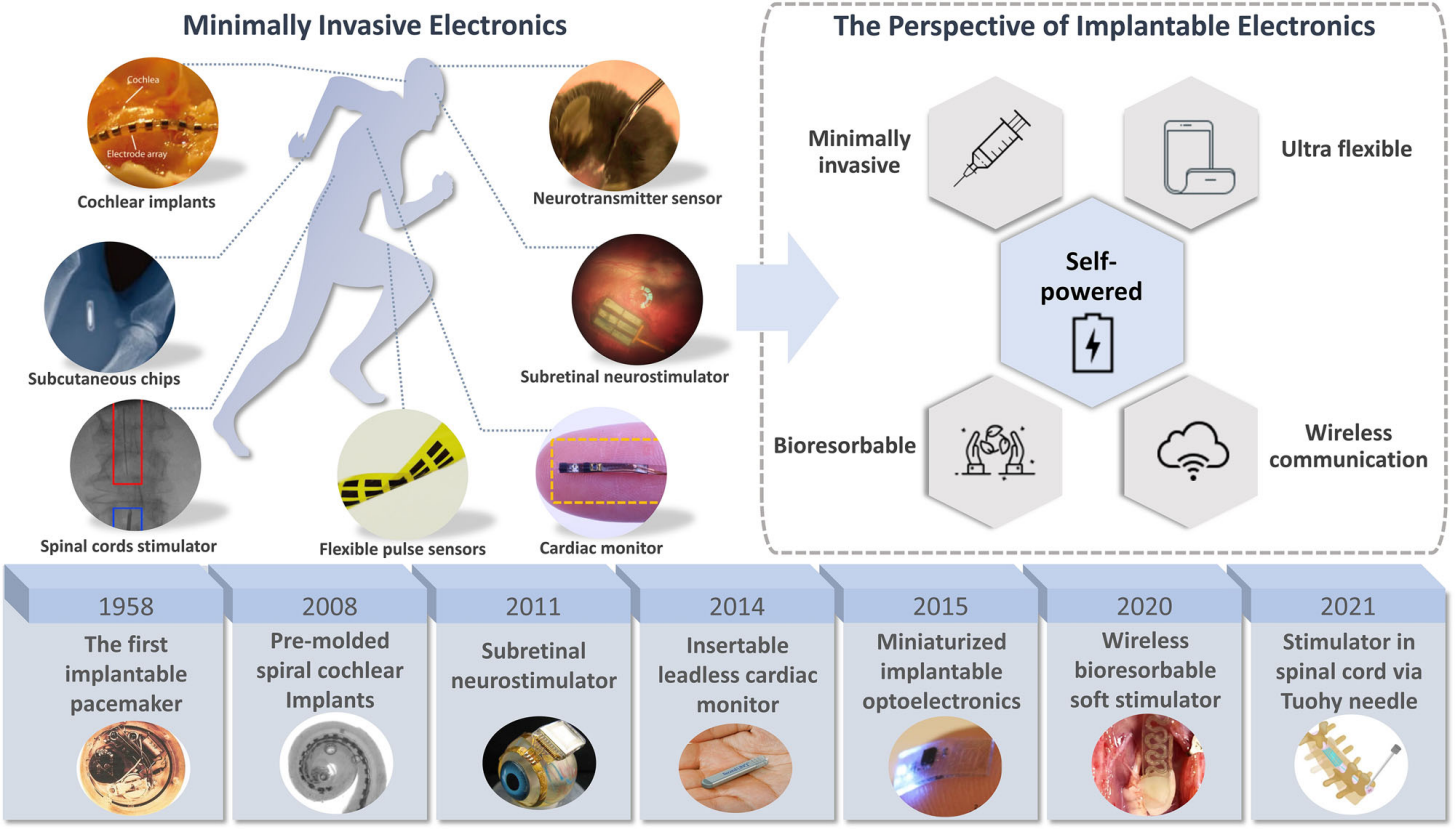
Milestones in the development history of implantable electronics, and an illustration of the advanced minimally invasive electronics implanted in various parts of the human body and perspective outlook for the features of implantable electronics in the future. Reproduced with permission.^[^ ^43^ ^]^ Copyright 2006, PubMed Central. Reproduced with permission.^[^ ^46^ ^]^ Copyright 2008, IEEE. Reproduced with permission.^[^ ^47^ ^]^ Copyright 2011, IEEE. Reproduced with permission. Copyright 2023, Medtronic. Reproduced with permission.^[^ ^39^ ^]^ Copyright 2015, Springer Nature. Reproduced with permission.^[^ ^24^ ^]^ Copyright 2020, Springer Nature. Reproduced with permission.^[^ ^52^ ^]^ Copyright 2021, Science. Reproduced with permission.^[^ ^53^ ^]^ Copyright 2023 Icons8 LLC. Copyright 2022, Springer Nature.^[^ ^55^ ^]^ Reproduced with permission.^[^ ^56^ ^]^ Copyright 2019, ARVO. Reproduced with permission.^[^ ^57^ ^]^ Copyright 2021, Science. Reproduced with permission.^[^ ^58^ ^]^ Copyright 2007, IEEE. Reproduced with permission.^[^ ^59^ ^]^ Copyright 2018, Wiley‐VCH GmbH.

Conventionally, implantable electronics with hardware modules such as bio‐functional parts, circuits and energy storage devices are packaged and sealed within bulky metal cases, then implanted into the vacant area of the human body by open surgery.^[^ ^42^ ^]^ Clinical implants such as drug delivery devices, pacemakers and ECG monitors are also in such conventional form. Figure 2 provides an overview of the milestones in the development history of implantable electronics since the first implantable battery‐powered cardiac pacemaker was invented in 1958.^[^ ^43^ ^]^ However, many complications including infection, thrombosis, lead failure and pneumothorax are related to this kind of construct of the cardiac pacemaker, the traditional implantation will cause large incisions and cannot exempt patients from secondary surgery to take implants out after the energy of the system is running out. Postsurgical complications caused by these invasive surgeries and bulky implants have also been reported.^[^ ^44^ ^]^ In this case, an implantable device with an ultrathin structure and good conformability will be a better choice due to the minimized volume and better matching with target organs. Take cochlear implant electrodes as an example, the first‐generation electrodes developed in the 1980s and 1990s demonstrated severe insertion trauma,^[^ ^45^ ^]^ and the improved design of configuration can help avoid the severity of the injury. A pre‐moulded spiral cochlear electrode implanted with the advanced off‐stylet technique can reduce contact with the outer wall of the scala tympani, soft top and silicone rubber surface is also helpful to prevent internal tissue trauma.^[^ ^46^ ^]^ Similarly, a hermetic vision prosthesis with a wireless‐operated subretinal neurostimulator has been developed and implanted in a minipig eye in 2011.^[^ ^47^ ^]^ To reduce the risk of infection caused by wireless transmission coils under the delicate conjunctiva, the coils were carefully wound on a spherical mandrel to match the curvature of the eye and moulded in biocompatible flexible poly(dimethylsiloxane) (PDMS) encapsulation. Another way to achieve minimally invasive implantation was developed in 2014 by Medtronic, a medical device company. A subcutaneous insertable cardiac monitor (the Reveal LinQ) with a high aspect ratio structure was designed for continuous cardiac monitor such as arrhythmia and atrial fibrillation, the parylene‐based encapsulation and minimal dimension (44.8 × 7.2 × 4.0 mm^3^) allow it to be inserted subcutaneously through an incision size less than 1 cm without any post suturing process.^[^ ^42^, ^48^ ^]^ With the programmable electronic system and lithium carbon monofluoride battery, the electronics system can be inserted into an infant's chest and work independently for up to 3 years with wireless data communication with medical centres.^[^ ^49^ ^]^ Even so, mechanically rigidness inducing stress to the interface between devices and tissue is still a nonnegligible issue, it is still necessary to seek a flexible and thin‐film configuration for such kinds of IMEs. Soft and stretchable materials with low modulus will be a desirable choice here. In 2015, Park et al. constructed a miniaturized, fully implantable soft optoelectronic system for wireless optogenetics consisting of radio frequency harvester antenna for wireless power and light‐emitting diode (LED) arrays to activate opsins.^[^ ^39^ ^]^ Inside the area of only 3 × 3 mm, the electrical interconnects and the circuits were sealed by flexible polyimide with thickness of 3 μm and low‐modulus silicone elastomer with thickness of 100 μm. This design enabled the implanted system to accommodate anatomical shapes and natural motions, and to achieve minimally invasive fully implantation on multiple neural interfaces.^[^ ^50^, ^51^ ^]^ Furthermore, soft and wireless powered bioresorbable electronic were developed by Choi et al. for the neuromuscular regeneration stimulator.^[^ ^24^ ^]^ Bioresorbable dynamic covalent polyurethane (b‐DCPU) was synthesized and applied as substrate and biofluid barrier for deformable filamentary serpentine interconnects, the elastomeric mechanics and low levels of swelling in biofluids enable the implants to serve for over 30 days with long‐term stability during the stimulation operation. With wireless power transmission technology and bioresorbable feature, the bioresorbable electronics can be implanted once for all and operate independently till full dissolution into benign products without residues, which avoids surgical extraction or secondary invasion for replacement. This kind of bioresorbable wireless powered electronics are also desirable options for bone healing treatment, spinal cord stimulation, brain therapy and cardiac pacing. Recently, minimally invasive spinal cord stimulation via simple needle puncture under local anaesthetic instead of traditional bulky paddle‐type devices requiring invasion under general anaesthetic have been reported.^[^ ^52^ ^]^ Since spinal cord implants are softer and soft robotics has made great progress regarding the surgical manipulators, Woodington et al. developed a minimally invasive spinal cord stimulation which can be loaded into needle for percutaneous implantation with low risk surgery.^[^ ^52^ ^]^ A 14‐gauge Tuohy needles were used for the loading and insertion of the rollable devices with dimension less than 2 mm including fluidic connections, electrical connections and supporting tubing. Due to the narrow footprint and flexibility, insertion trauma can be reduced, and on‐demand shape actuation can be achieved. Compared with the previous rigid devices, it can offer far fewer surgical risks with simple epidural needle insertion. Till now, huge progress has been made towards the minimally invasive designation of implantable electronics as can be seen from the milestones in the development history.

Comparatively, biomedical electronics with a high aspect ratio structure such as a needle‐like shape or integrated on a tubular carrier can solve the problems of a large incision, and enables accurate and minimally invasive injection of biomedical electronics into inhomogeneous tissues.^[^ ^51^ ^]^ Nowadays, injectable biomedical devices were widely studied and applied in the field of brain sciences, percutaneous therapies, reading vital clinical signs, and healing diseases et al.^[^ ^42^ ^]^ With a high aspect ratio form, injectable biomedical devices with various designs can be applied to almost every part of the human body. As illustrated in Figure 2 top left, various state‐of‐the‐art minimally invasive electronics were developed and implanted in different parts of human body. For instance, tissue‐like neurotransmitter sensor for neuronal recording in the brain and gut with minimal damage to other regions termed NeuroString were designed by Li et al.^[^ ^53^ ^]^ As tissue‐mimicking stretchable neurochemical biological interface sensors, it enables long‐term in vivo real‐time sensing in the brain and simultaneously serotonin dynamics detecting in the gut of a behaving mouse with high data recording fidelity. With easily stretched, twisted and even knotted structures, the ultra‐flexible NeuroString can get access to the twisting colon of a mouse with no subsequent insertion trauma representing its high compatibility with soft and complex tissues. It is expected to be applicable for non‐invasive biomolecular monitoring and dynamic signal study throughout the body in primates. The cochlear implant is another successful electronic prosthesis affecting millions of people worldwide, it changed the lives of people with profound hearing loss by long cable‐like electrodes implanted into the spiral‐shape cochlear area for hearing aid and restoration.^[^ ^46^, ^54^ ^]^ According to Pinyon et al., cochlear implant electrode array integrated with neurotrophin gene therapy can produce directed regeneration of spiral ganglion neurite through stimulation in the guinea pig.^[^ ^55^ ^]^ It provides minimal extraneous electrical stimulation for auditory nerve regeneration, nerve fibre can be restored to the pre‐deafness values. Subretinal neurostimulators as a novel technology can also help individuals suffering from retinitis pigmentosa and macular degeneration to restore useful vision. The reported neurostimulation array based on a soft polyimide substrate was constructed and inserted through the scleral incision into the subretinal space with only a 3 mm wide incision.^[^ ^56^ ^]^ After successful implantation, excellent retina apposition and intact inner layers of the overlying retina can be retained. For the cardiovascular system to deliver oxygen and nutrients to the body, implantable catheter‐type oximeters can help to provide accurate real‐time monitoring of vascular oxygen saturations. Compared with existing glass fibre‐optic catheters leading to blood vessel damage and infection, the recently reported miniaturized wireless optoelectronic catheter system is more patient‐friendly with good flexible construction and the absence of physical tethers, and especially, the diameter of the probe is only 1.5 mm.^[^ ^57^ ^]^ With the addition of wireless data transmission, real‐time local tissue oxygenation and respiratory activity can be monitored during continuous operation with accuracy and precision at clinical standards. Injectable subcutaneous chips like radiofrequency identification (RFID) tags in subcutaneous areas of the human body for recording and tracking personal healthcare information have also been reported.^[^ ^58^ ^]^ Through a 2 mm incision, a mini transmitter about the size of a grain of rice can be implanted subcutaneously for the control of doors, lights, and computers, and can also provide personal medical information when patients are in emergency unconscious situations. Under the subcutaneous area, soft and flexible configurations can further alleviate irritations and chronic damage to surrounding tissues with enhanced mechanical compatibility with soft skin. To realize the minimally invasive subcutaneous implantation, a flexible‐device injector was reported and an ultra‐flexible optical pulse sensor was successfully implanted into a live pig animal model with the injector via a small incision of 4 mm.^[^ ^59^ ^]^ Finally, spinal cord implants for neuronal activity measurement and stimulation as the oldest and most established neuromodulation therapeutic electronics have now developed towards injectable minimally invasive implants via simple needle puncture compared with traditional bulky devices.^[^ ^52^ ^]^ Though in various forms and with different advantageous functions, these injectable biomedical devices are designed to be injected into aimed sites inside organisms with minimal invasions.

Enormous advancements and continuous breakthroughs have been achieved in implantable and injectable biomedical electronics, which hugely advanced the quality of surgical and monitoring tools. Besides the gradually minimized dimension of the implantable bioelectronic, the barrier hermeticity has also been widely studied especially on the advanced thin film encapsulation and packaging coating approaches.^[^ ^60^ ^]^ Long‐term reliability of next‐generation biomedical implants highly relies on new packaging solutions which solve the isolation and biocompatibility problems of chronic flexible implants in harsh environments with biofluids and irregular geometrical constraints. The discussion of strategic materials selection including natural polymers, synthesized polymer and inorganic materials can also provide guidelines to design IMEs in various application scenery with diversified demands (e.g. instant or on‐demand bio absorbability, stretchability, transparency, breathability, self‐healing, water permeation blocking etc.).^[^ ^61^, ^62^ ^]^ Based on the state‐of‐the‐art advancements, the future development tendency of the advanced biomedical implantable electronic system will shift towards minimally invasive, ultra‐flexible, bioresorbable, wireless and multifunctional to achieve more pain‐free surgical implantation and high‐accuracy bio functional monitoring.

## 3 MINIMALLY INVASIVE POWER SOURCES

Commonly a power source is contained in a fully implanted biomedical device, the demand for minimally invasive power sources will continue to increase as the minimally invasive bioelectronic applications have been well developed and will expand rapidly.^[^ ^8^ ^]^ The commonly used power source for IMEs is bulky electrochemical power sources such as batteries and supercapacitors due to the mature technology and available hardware.^[^ ^63^, ^64^ ^]^ Conventional lithium‐ion batteries can meet the requirement of energy supply due to their reliability and high energy density (≈400 Wh kg^−1^).^[^ ^15^, ^65^ ^]^ Nevertheless, lithium‐ion batteries in the bulky, rigid and large‐size form will not only result in a large total volume of the implant system but also nonnegligible issues such as irritation and infections after surgery. Additionally, issues such as battery replacement after charge depleting, toxic material leakage of electrodes and electrolytes, and rigid shape mismatched with 3D dynamically curved tissues may lead to the unprecise interpretation of experimental data.^[^ ^17^, ^35^ ^]^ Obviously, the dimension and lifespan of power sources represent the major limiting factors for the development of the advanced biomedical implantable electronic system at present. Therefore, a qualified implantable power source for novel biomedical implants should possess the following features: miniaturized, light‐weight, adequate capacity, and mechanically deformable properties that match well with soft biological organs and tissues, composed of biocompatible materials.^[^ ^16^ ^]^ Since the power source plays a crucial part in the development of IMEs, many efforts and enormous enthusiasm of researchers have been dedicated to developing implantable power sources to extend the lifespan of IMEs.^[^ ^66^, ^67^ ^]^ With different functions and diagnostic purposes, IMEs show different requirements for the power source. For short‐term applications, biodegradable batteries with no need for secondary surgery will be a desirable choice.^[^ ^68^ ^]^ For long‐term applications, a power source with enhanced capacity such as batteries or energy source harvesting power wirelessly in‐vivo and from the external of the human body will be more suitable. There are many forms of energy sources optional for IMEs such as thermal, kinetic, biochemical, electromagnetic, acoustic, and radiative forms of energy.^[^ ^16^ ^]^ For example, energy derived from the human body including glucose oxidation,^[^ ^69^ ^]^ heartbeats, and organ motion,^[^ ^70^, ^71^ ^]^ can be harvested by biofuel cells,^[^ ^72^ ^]^ and piezoelectric/triboelectric energy harvesters.^[^ ^73^, ^74^ ^]^ Also, wireless power transmission technologies including inductive coupling/RF,^[^ ^75^ ^]^ photovoltaic,^[^ ^76^ ^]^ and ultrasound‐induced power transfer^[^ ^77^ ^]^ can be used as direct power or to assist in recharging the existing energy storage devices. Benefiting from these energy solutions, self‐powered IMEs can be realized. To further discuss the practical application of different minimally invasive power sources based on the above mechanisms to power the IMEs, three categories were summarized: Energy storage devices (e.g. biodegradable primary batteries, rechargeable batteries, fibre supercapacitors), human body energy harvester (e.g. PENG, TENG, biofuel cell) and wireless power transfer (e.g. near‐field magnetic resonant coupling, far‐field RF radiation, PV power transfer, ultrasonic power transfer). Application contexts and advantages of each minimally invasive power source will be discussed in detail.

### 3.1 Energy storage devices

#### 3.1.1 Biodegradable primary batteries

Batteries, including the single‐use primary battery and rechargeable secondary battery, have been developed and can provide sufficient energy for IMEs with a lifespan of years.^[^ ^65^ ^]^ Various IMEs including neurostimulators, cardiac pacemakers and cardiac defibrillators have applied batteries as energy sources. Not like batteries in other electronics, implantable batteries need to satisfy strict and considerable requirements including high energy density, stability, complete packaging, low self‐discharge and a battery life of years to not only support vital functions in human bodies but also ensure the safe operation of devices. Also, small volumes and lightweight are required in the limited space inside the human body. Li‐CF*_x_* (lithium‐carbon monofluoride) and Li‐SVO (lithium‐silver vanadium oxide) batteries have been applied in the industry for cardiology applications for decades.^[^ ^65^ ^]^ As an example, the Reveal LinQ insertable cardiac monitor fabricated by Medtronic in 2014 is a wireless and powerful small insertable cardiac monitor ideal for patients experiencing infrequent symptoms that require long‐term monitoring or ongoing management, the device consists of two titanium nitride‐based recording electrodes, programmable electronic system and a Li‐CF*_x_* battery that powers the entire system up to 3 years.^[^ ^48^ ^]^ Except for Li‐CF*_x_* ^[^ ^78^ ^]^ and Li‐SVO batteries,^[^ ^79^ ^]^ lithium–iodine (Li–I_2_)^[^ ^80^ ^]^ and lithium manganese dioxide (Li–MnO_2_)^[^ ^81^ ^]^ have also been developed and widely applied in IMEs.

**FIGURE 3:**
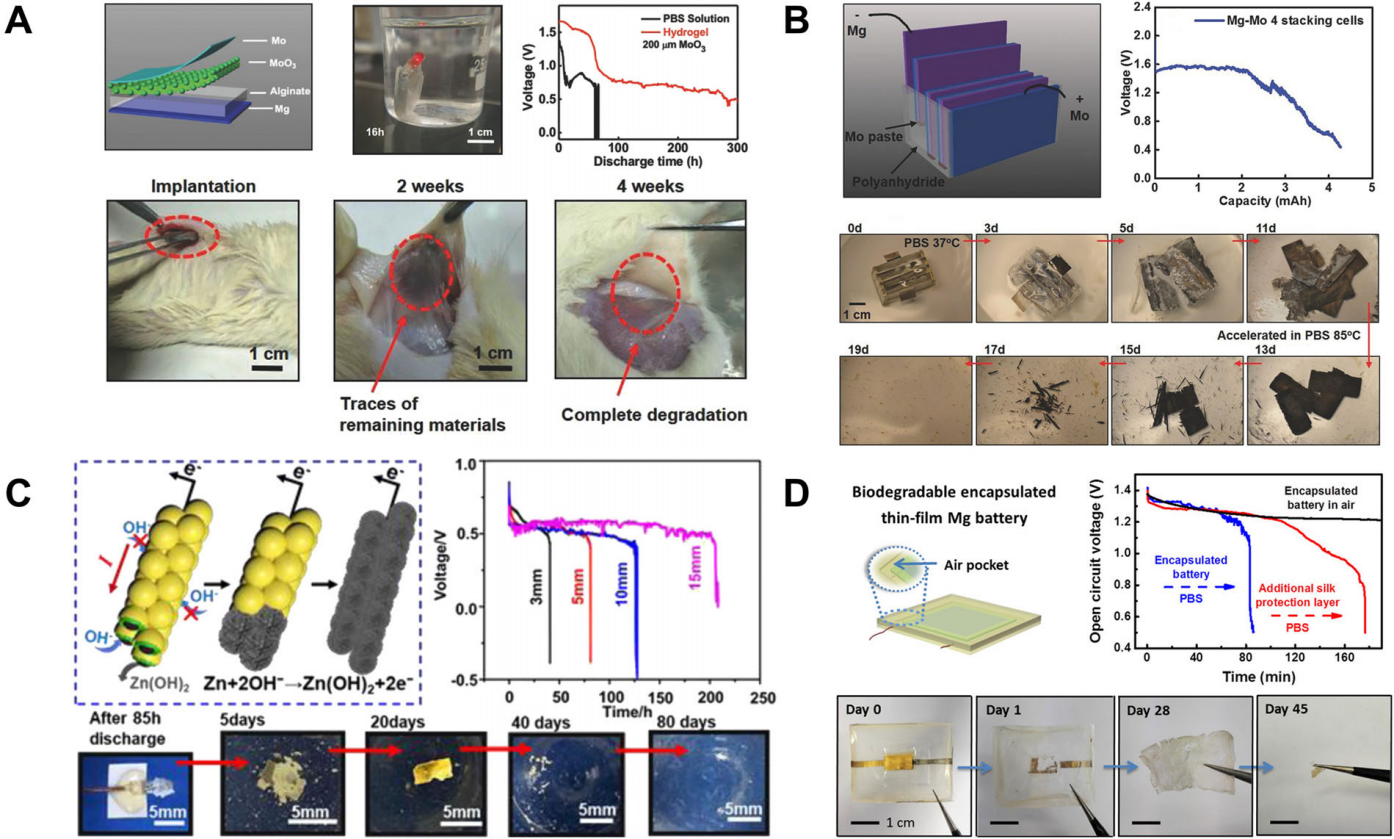
Biodegradable primary batteries. (A) Biodegradable Mg–MoO_3_ primary battery works for about 13 days. Reproduced with permission.^[^ ^22^ ^]^ Copyright 2018, Wiley‐VCH GmbH. (B) Fully biodegradable Mg–Mo primary batteries. Reproduced with permission.^[^ ^19^ ^]^ Copyright 2014, Wiley‐VCH GmbH. (C) Bioresorbable zinc primary battery with zinc microparticle network coated with chitosan and Al_2_O_3_ double shells as the anode. Reproduced with permission.^[^ ^20^ ^]^ Copyright 2021, American Chemical Society. (D) Biodegradable thin film Mg primary battery with silk fibroin‐IL electrolyte. Reproduced with permission.^[^ ^21^ ^]^ Copyright 2017, American Chemical Society.

At the same time, the primary batteries have limitations such as rigid structure, capacity loss and battery failure, and therefore cause non‐healing incisions as well as the risk of complications after insertion. With the emerging transient electronics technology, an alternative strategy is to develop biocompatible or biodegradable batteries.^[^ ^82^, ^83^, ^84^, ^85^ ^]^ Considering the safety issue which might be caused by the leakage of the metal‐based material, metals like Mg and Zn are safer than Li inside the human body. In 2014, Yin et al. first reported fully biodegradable batteries consisting of dissolvable Mg‐X (X = Fe, W, or Mo) foils as anodes and cathodes, and polyanhydrides for encapsulation. For short‐ and medium‐term applications, biodegradable features are desirable so that a second surgical removal is not required.^[^ ^16^ ^]^ Recently, Mg‐ and Zn‐based batteries have made great progress. For instance, Mg and its alloys with high theoretical capacity (2.2 Ah g^−1^) are promising anode materials and represent excellent biocompatibility (daily allowance ≈300 mg d^−1^).^[^ ^22^ ^]^ According to Huang et al., they designed a completely biodegradable primary magnesium‐molybdenum trioxide (Mg–MoO_3_) battery with high performance as illustrated in Figure 3A. In their design, a single‐cell battery with a stable output voltage (1.6 V) can achieve a high energy density of 6.5 mWh cm^−^^2^. The battery can work for about 13 days after encapsulation with biodegradable polyanhydride and poly(lactide‐*co*‐glycolide) (PLGA), and complete biodegradability of the battery can be observed in vivo experiments. With stable output voltage and a relatively long lifespan, this battery system could meet the requirements of short‐term/medium‐term and implantable electronics requiring ultralow power, which represents a promising method for independent battery‐powered IMEs. Similarly, Yin et al. reported a water‐activated primary batteries with magnesium foils as anodes and metal foils based on Fe, W, or Mo as the cathodes.^[^ ^19^ ^]^ The primary battery was encapsulated by biodegradable polyanhydrides, it can provide a stable voltage output of about 1.6 V for up to 6 h when discharging at a current density of 0.1 mA cm^−^^2^ as shown in Figure 3B. From the degradation process, it can be observed that the polyanhydride cover dissolved first followed by the degradation of Mg and Mo foils in phosphate‐buffered saline (PBS) solution at 37°C, and the primary battery was fully degraded after 19 days by increasing the temperature to 85°C. Based on their transient batteries, the minimum dimension required to power the wireless implantable sensing is 5 mm with a thickness of 3.46 mm for operation for 1 year, which is a fully biocompatible and environmentally benign power source for IMEs. In addition, zinc primary battery is another option while Zn metal has a slower degradation rate, it can avoid local pH increases and alleviate the evolution of gaseous hydrogen. According to Dong et al., a bioresorbable zinc primary battery was constructed with zinc microparticle network coated with chitosan and Al_2_O_3_ double shells as the anode.^[^ ^20^ ^]^ When discharged at a current of 0.01 mA in saline, the battery can generate stable voltage output of 0.55 V with a cross‐sectional area of only 0.17 × 2 mm^2^. With 15 mm Zn anode, the battery can discharge stably for 200 h and can be fully dissolved in the saline solution, as can be observed in Figure 3C, indicating a significant potential for in vivo powering transient IMEs. In some cases, ionic liquids (ILs) with stable potential window and high ionic conductivity can be utilized as additives for biopolymers and biocompatible electrolytes.^[^ ^86^ ^]^ As Jia et al., demonstrated, a biodegradable thin film magnesium primary battery was designed with biocompatible IL (choline nitrate) and ion‐conducting free‐standing membrane of biodegradable silk fibroin.^[^ ^21^ ^]^ The silk protection layer can help protect and control degradation. With another layer of silk fibroin, stable open‐circuit voltages above 1.21 V for more than 100 min can be observed and then finally be fully decomposed as can be seen in Figure 3D.

At present, bioresorbable electronics or transient electronics have been widely studied and have now achieved significant progress in mechanism and applications, it becomes an emerging technology in the field of innovative healthcare electronics.^[^ ^87^ ^]^ Benefiting from the existing bioresorbable materials (e.g. Mg, Zn, Mo, polylactic acid, polyglycolic acid, polylactic‐co‐glycolic acid, carbon nanotubes, graphene),^[^ ^88^, ^89^, ^90^, ^91^, ^92^ ^]^ biodegradable primary batteries can provide power for the bioelectronic functions such as post‐surgical monitoring of organ, tissue, and wound healing for a period from seconds to months, and finally fully decomposed into biological safe byproducts through hydrolysis and enzymatic degradation, which significantly reduced the risk of irritations and complications caused by surgery. It is high expected to lead a revolutionary change in the field of power sources for minimally invasive IMEs, while in the meantime, there are some critical challenges including the high‐cost fabrication techniques, uncontrollable packaging methods, and the dissolution mechanisms of bioresorbable electronics still exist and require further exploration.

#### 3.1.2 Rechargeable batteries

**FIGURE 4:**
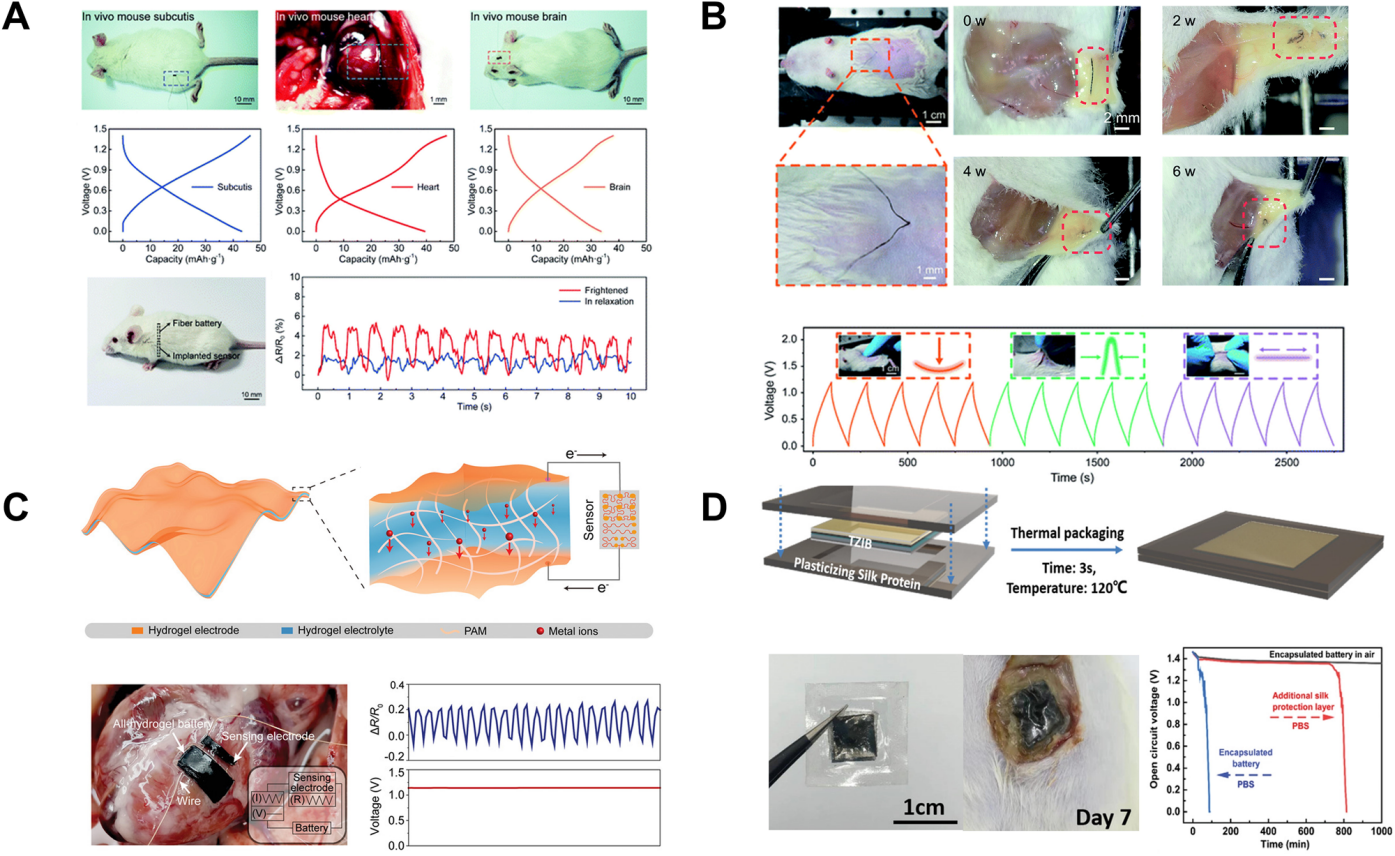
Miniaturized rechargeable batteries with one‐dimensional and coplanar configuration. (A) Injectable fibre sodium‐ion battery for all‐region power supply in vivo. Reproduced with permission.^[^ ^26^ ^]^ Copyright 2021, Royal Society of Chemistry. (B) Biodegradable and rechargeable sodium‐ion batteries with fibre configuration. Reproduced with permission.^[^ ^25^ ^]^ Copyright 2021, Royal Society of Chemistry. (C) A tissue‐like soft all‐hydrogel battery powering a hydrogel strain sensor for the heartbeat monitor. Reproduced with permission.^[^ ^93^ ^]^ Copyright 2022, Wiley‐VCH GmbH. (D) Biodegradable rechargeable zinc ion battery with controlled degradation and stable electrochemical performance. Reproduced with permission.^[^ ^94^ ^]^ Copyright 2022, Wiley‐VCH GmbH.

Compared with the single‐used primary battery, rechargeable batteries are still a compelling topic in the future research of IMEs energy storage devices since they can be recharged after the first implantation and provide long‐term stable power in vivo without the need for replacement surgery. With one‐dimensional structures and miniaturized coplanar configuration, rechargeable batteries are more beneficial for minimally invasive IMEs application and enable the implanted system to work with stable power sources. Recently, Peng et al. designed a one‐dimensional fibre‐like rechargeable aqueous sodium‐ion battery that can be injected into different parts of the body for energy supply as can be seen in Figure 4A.^[^ ^26^ ^]^ With aligned carbon nanotube (CNT)/Na_0.44_MnO_2_ (NMO) hybrid fibre as cathode and CNT/molybdenum trioxide/polypyrrole (CNT/MoO_3_/PPy) hybrid fibre as anode, good mechanical and electrochemical performance were achieved. With the remarkable one‐dimensional structure, the fibre sodium ion battery possessed advantages including good flexibility and minimized implantation incision size. Also, the fibre battery is exposed to biofluid which acts as electrolyte and can be injected into tissue directly. Due to the fibre structure with high flexibility, the battery can match the soft tissue well and achieve stable interface contact. The electrochemical performance of the fibre battery with a power density of 78.9 mW cm^−^^3^ can power most of the biomedical applications in vivo including the implanted sensor for respiration monitoring.

Combined with bioresorbable features, the syringe‐assisted injectable bioresorbable fibre battery can provide a better solution for the non‐invasive implantation of electronics with low health risks. For example, Mei et al. demonstrated a biocompatible aqueous sodium‐ion battery that can be injected into the body without immune responses and degraded after completing the mission.^[^ ^25^ ^]^ They utilized biodegradable materials to construct the fibre battery, the polydopamine/polypyrrole composite as anode and MnO_2_ as the cathode was coated on the conducting fibre and the biodegradable chitosan was applied as the separator. After twisting the electrodes fibre together and injecting it into the abdominal subcutis of an experimental mouse, with the body fluid acting as the electrolyte, the fibre battery showed a specific capacity of 25.6 mAh g^−1^, and a good cycling stability with retention of 69.1% after 200 cycles. As demonstrated as a power source for the biosensor, the fibre battery succeeds in detecting the pressure changes in the implanted area and is biodegraded as designed through hydrolysis and enzymolysis. The flexible battery also showed excellent stability during the pressing, bending and stretching as can be observed in Figure 4B, the biodegradable fibre sodium‐ion battery was finally fully biodegraded after several weeks avoiding the need for surgery for removal. The minimized injectable batteries are very promising choices for IMEs due to their better biocompatibility, longer service time and better stability inside the human body. With a coplanar structure, the ultrasoft rechargeable lithium‐ion/zinc‐ion batteries with low Young's modulus were exploited. Ye et al. proposed tissue‐like ultra‐soft all‐hydrogel lithium‐ion/zinc‐ion batteries.^[^ ^93^ ^]^ With the interfacial dry crosslinking strategy, superb electrical conductivity and high interface charge transfer efficiency were achieved. Benefiting from this strategy, the lithium‐ion battery‐based all‐hydrogel battery achieved high specific capacities of 82 mAh g^−1^ and the all‐hydrogel zinc‐ion batteries represented 370 mAh g^−1^ at a current density of 0.5 A g^−1^. While integrated with a hydrogel strain sensor, the all‐hydrogel battery can supply a stable power output for the heartbeat monitor while precisely detecting the strain change (Figure 4C). In addition, a biodegradable rechargeable zinc ion battery with controlled degradation and stable electrochemical performance was reported.^[^ ^94^ ^]^ In this battery, a biodegradable cellulose aerogel–gelatin solid electrolyte was designed, a biodegradable highly flexible silk protein film was used as passivation, and the zinc thin‐film battery achieved a stable output voltage of up to 1.6 V. After implantation, the degradation in the subcutaneous area of rats can be observed in Figure 4D. The complete degradation of this thin‐film battery in vivo demonstrated a non‐toxic and harmless implantation procedure to the hosts (rats) and an advanced power source for clinical electronics.

Compared with primary batteries, rechargeable batteries offer a more durable energy storage strategy for IMEs without the need of replacement by open surgery after implantation and can support the implanted electronic system for a longer service period. In this case, a long‐term cycling stability is expected for implantable rechargeable batteries. Supercapacitors with long cycling life span beyond 10 thousand cycles therefore would be another complementary to support stable operation. Also, the rechargeable battery‐powered systems based on tethers such as charging wires with redundant cable connection still pose a limitation to the development of IMEs in the future. The combination of minimized implantable rechargeable batteries or supercapacitors with wireless charging technologies as well as controllable biodegradation in desired service time will benefit the post‐processing after implantation, and therefore is worthy of expectation for the self‐powered IMEs system in the near future.

#### 3.1.3 Supercapacitors

**FIGURE 5:**
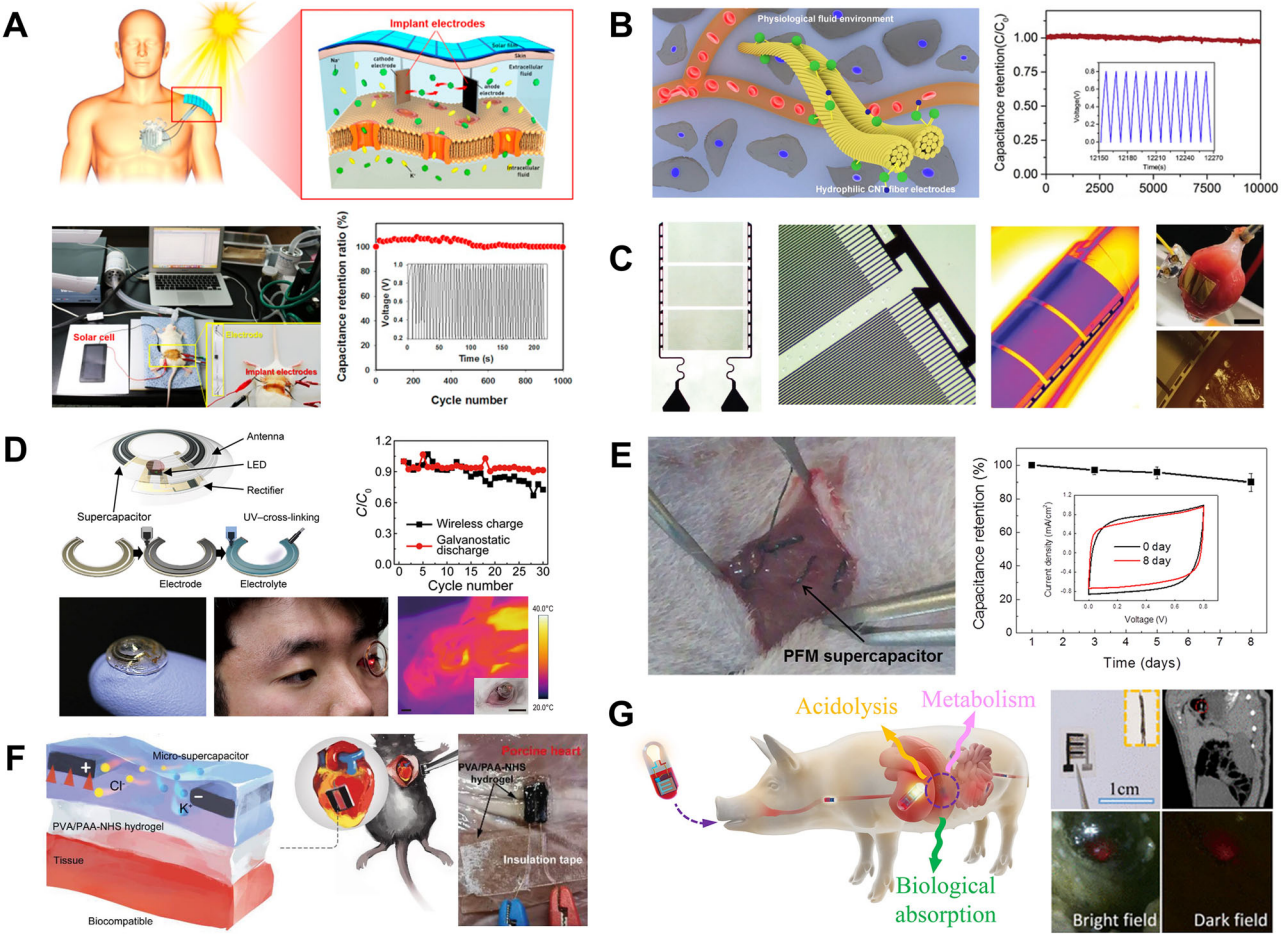
Miniaturized supercapacitors for implantation and electrical modulation of bio‐interfaces. (A) A biocompatible supercapacitor based on body fluids as electrolyte combined with solar cells for energy harvesting and storage. Reproduced with permission.^[^ ^102^ ^]^ Copyright 2017, Elsevier. (B) A miniaturized implantable supercapacitor based on a biocompatible aligned carbon nanotube fibre electrode in PBS. Reproduced with permission.^[^ ^103^ ^]^ Copyright 2017, Elsevier. (C) A carbon‐based flexible micro‐supercapacitor system for various types of bioelectronic interfaces (scale bar on the top right, 5 mm). Reproduced with permission.^[^ ^104^ ^]^ Copyright 2020, Springer Nature. (D) Solid‐state supercapacitors powering the flexible smart contact lenses wirelessly. Reproduced with permission.^[^ ^105^ ^]^ Copyright 2019, Science. (E) A biomolecule‐based fibre‐type implantable supercapacitor with micro diameter for in vivo energy storage. Reproduced with permission.^[^ ^27^ ^]^ Copyright 2018, Elsevier. (F) Biocompatible wet‐adhesive all‐hydrogel supercapacitor with good performance and stretchability on the heart of mice. Reproduced with permission.^[^ ^106^ ^]^ Copyright 2021,Wiley‐VCH GmbH. (G) An ingestible and nutritive zinc‐ion micro‐supercapacitor in porcine stomach. Reproduced with permission.^[^ ^107^ ^]^ Copyright 2022, American Chemical Society.

With fast charging and discharging rates, high power density (>10 kW kg^−1^), and splendid cycling lifespan (>100,000 times), supercapacitor bridged the gap between electrolytic capacitors and rechargeable batteries. Supercapacitors and emerging hybrid ion capacitors with increased energy density without deteriorating power density arouse intensive research interest.^[^ ^95^ ^]^ Such kind of energy storage devices can act well as an energy reservoir for electronics when unexpected interruptions in the power source occur, which showed exceptional rate capability and good stability.^[^ ^96^, ^97^ ^]^ Depending on different mechanisms, supercapacitors can be divided into two types. One is electrical double‐layer capacitance (EDLC) relying on ionic electrochemical adsorption/desorption at the electrode/electrolyte interfaces, another is pseudo‐capacitors depending on pseudo‐capacitance from rapid redox reactions occurring on (or near) electrode surfaces. Due to the uncertainty of the pathological characteristics of most biocompatible materials, and the discontinuity of electrode elements under the complex and variable surface strain, new degradable energy devices often show unpredictable performance and huge safety risks in the diagnosis process. Therefore, with minimized dimension,^[^ ^98^ ^]^ good flexibility and strong selectivity of non‐toxic electrode materials, microscale supercapacitors have a bright application prospect in the area of medical auxiliary energy sources.^[^ ^99^ ^]^ At present, wearable devices usually use micro‐batteries and micro‐supercapacitors as energy supply and have the potential to be integrated with electronic devices in a small size. For microelectronics requiring long‐term service and high‐power density, micro supercapacitors would be a good choice and therefore have drawn wide attention. In the field of biomedical electronics and sensors, micro‐batteries as energy supply inevitably suffer from frequent replacement due to the short cycling life of about several thousand times. Comparatively, micro supercapacitors represent excellent cycling stability with negligible capacitance decay, which effectively avoids the frequent replacement issue. Also, unlike micro batteries, micro supercapacitors with higher power density with small volumes are more suitable for flexible IMEs requiring high power density than batteries requiring additional integration to achieve high power density.^[^ ^100^, ^101^ ^]^ However, conventional supercapacitors also suffer from rigid and heavy structures, unstable electrolytes and rigid sealing before implantation, which is unfit for IMEs. Some researchers reported strategies that body fluid was used as electrolytes without encapsulation. According to Chae et al., a conceptual system adopts solar cells as energy supply and supercapacitor as energy reservoir, MnO_2_ and carbon were used as electrodes inserted into the subcutaneous area of a rat, and biofluids were used as electrolytes (Figure 5A).^[^ ^102^ ^]^ The working voltage window of the implanted supercapacitor is from 0.2 to 1.0 V and good cycling stability was achieved with over 1000 cycles. Similarly, as shown in Figure 5B, with biocompatible carbon nanotube fibres as electrode material and biofluid as the electrolyte, He et al. fabricated supercapacitors without additional encapsulation, a specific capacitance of 10.4 F cm^−^^3^ and long cycling stability (98.3% retention after 10,000 cycles in PBS) were achieved.^[^ ^103^ ^]^ Furthermore, for the electrical modulation of tissues and organs, bio‐interfaces at the macroscopic level are in demand. Fang et al. fabricated a flexible micro supercapacitor‐like system with micelle‐enabled self‐assembly approach for various types of bioelectronic interfaces, as shown in Figure 5C. With interdigitated electrode design, the micro supercapacitors show a smaller size and are more suitable for subcellular interfaces. With this flexible micro supercapacitor‐like electronic system, multifunction was achieved including modulation of cardiomyocytes in vitro, excitation of isolated heart and retinal tissues ex vivo, stimulation of sciatic nerves in vivo, and bioelectronic cardiac recording.^[^ ^104^ ^]^ More than that, micro supercapacitors have also been applied to assist wireless power transfer for the continuous operation of electronic devices such as smart contact lenses due to the long cycling lifespan and high‐power density. With a wireless charging antenna continuously receiving external inductive power, the integrated micro supercapacitors can store energy and enable independent operation of the smart electronic device inside the human body. Park et al. reported a soft smart contact lens integrated with an antenna for wireless recharging and a solid‐state supercapacitor for energy storage.^[^ ^105^ ^]^ The monolithically integrated solid‐state supercapacitors showed excellent cycling stability up to 10,000 cycles and a stable temperature was maintained during wireless operation which ensured the safety of the wearer's eyes as illustrated in Figure 5D. Furthermore, fibre supercapacitor with high aspect ratio structure and micro diameter was constructed and applied for surgical suture for wound stitching and implantation in the blood vessel.^[^ ^27^ ^]^ The highly flexible and conductive electrode fibres was fabricated with biocompatible poly (3,4‐ethylenedioxythiophene): poly (styrenesulfonate) (PEDOT:PSS)/ ferritin nanoclusters trapped multiwalled carbon nanotube (MWNT) sheets, which enabled the fibre supercapacitor to operate well in a mouse with excellent biocompatibility. An areal capacitance of 32.9 mF cm^−^^2^ in PBS solution was achieved and above 90% of capacitance was maintained in the mouse after 8 days implantation as illustrated in Figure 5E. As supplementary, stitching the devices into fragile and curvilinear tissue area may cause damage and dehiscence, tissue‐adhesive implants are more desirable in this case.^[^ ^106^ ^]^ A biocompatible wet‐adhesive all‐hydrogel micro‐supercapacitor with good stretchability was designed and implanted onto the heart of living mice for 14 days as shown in Figure 5F. Polyaniline mixed with a composite of reduced graphene oxide and Mxenes was adopted as gel electrodes, the all‐solid hydrogel electrolyte can also act as the packaging material and boost the integration of the all‐solid micro‐supercapacitor. A desirable energy storage performance was also achieved with a high areal capacitance of 45.62 F g^−1^ and energy density of 333 μWh cm^−^^2^ while the Masson immunostaining of heart tissues shows that the adhering of micro‐supercapacitors causes no harm to cardiomyocytes and mice after 14 days’ implantation. In another implantation scene, an ingestible and nutritive zinc‐ion micro‐supercapacitor was reported.^[^ ^107^ ^]^ The micro supercapacitors can be loaded in a standard‐sized capsule and enter the porcine stomach through ingestion. While the composition of capsule shell is gelatin which is easily decomposed after entering the stomach, the edible electrode materials including active carbon and zinc can be exposed in acidolysis and be removed through metabolism without the need for retrieving (Figure 5G). The micro supercapacitors showed a high energy density of 215.1 μWh cm^−^^2^, it can power a red LED and can act as a nutritional supplement of zinc after digestion.

With the miniaturized energy storage devices, the viability of green, safe, and nontoxic in vivo detection could be considerably improved. Though the supercapacitor suffers from low energy density, the properties including high biocompatibility, long‐term safety, good cycling stability and high‐power density are highly desirable as an assistant energy storage device. Also, the various microfabrication technologies enable the various applicability of micro supercapacitors as micro implantable energy storage devices in every part of the living body. It is promising that the miniaturized supercapacitors coupling the wireless charging technologies will support the sustainable long‐term service of the IMEs system.

### 3.2 Human body energy harvester

Many forms of energy contained in the living organism, including chemical energy from the reaction of organic molecules such as glucose oxidation and mechanical energy from such as heart beating and respiration, have been widely studied for energy harvesting in vivo from the human body. The commonly studied internal energy‐harvesting devices include the nanogenerator (NG) converting mechanical energy (cardiovascular, respiratory and gastrointestinal) into electrical energy by piezoelectric and triboelectric effects, and another kind of harvester is biofuel cells generating energy from glucose oxidation. With these permanent energy sources in the human body, the implanted energy harvesters can be implanted once for all and work for a long period of time enough to support the electronics to finish the missions during the lifetime of the hosts, which is a promising method to reduce the pain and incision caused by a repeated second surgery.

#### 3.2.1 Nanogenerators (NGs)

**FIGURE 6:**
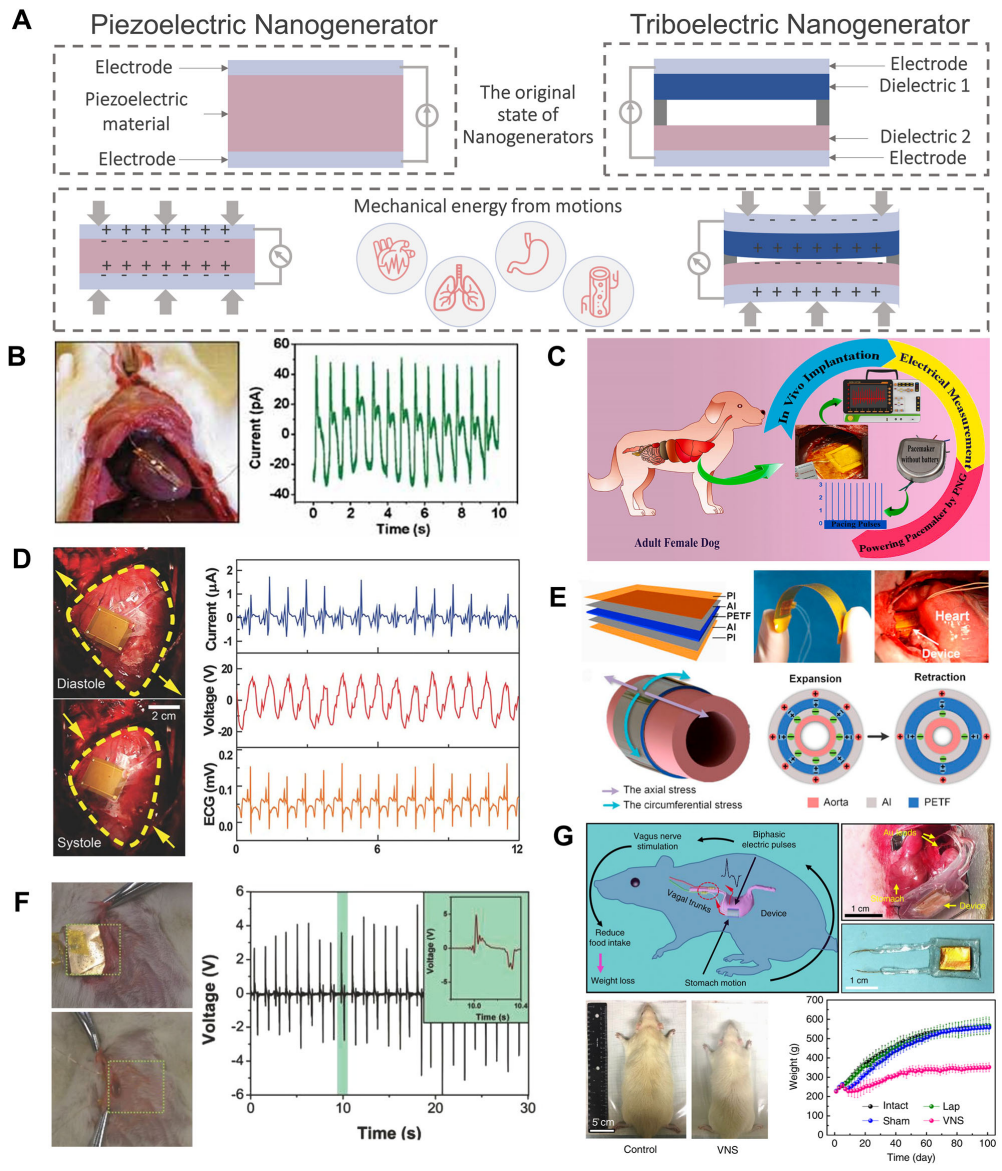
Implantable PENG and TENG energy harvesters. (A) Schematics of the electricity generation of the PENG and TENG harvesting energy from mechanical energy of organ motions. (B) An alternating‐current nanogenerator based on a single piezoelectric fine nanowire harvesting mechanical energy from the breath and heartbeat of a live rat. Reproduced with permission.^[^ ^111^ ^]^ Copyright 2010, Wiley‐VCH GmbH. (C) A self‐powered cardiac pacemaker by biocompatible polymer‐based PENG on the heart of an adult dog. Reproduced with permission.^[^ ^29^ ^]^ Copyright 2021, Elsevier. (D) In vivo simultaneously ECG signals recorded by flexible PENG attached to a porcine heart harvesting energy from rhythmical cardiac contraction and relaxation. Reproduced with permission.^[^ ^113^ ^]^ Copyright 2017, Wiley‐VCH GmbH. (E) Implantable thin film PENG powered in vivo blood pressure monitoring. Reproduced with permission.^[^ ^114^ ^]^ Copyright 2016, Elsevier. (F) Self‐powered cardiac pacemaker with breath driven implanted TENG in vivo. Reproduced with permission.^[^ ^30^ ^]^ Copyright 2014, Wiley‐VCH GmbH. (G) VNS device for effective weight control powered by TENG harvesting mechanical energy from gastric peristalsis. Reproduced with permission.^[^ ^119^ ^]^ Copyright 2018, Springer Nature.

Recently, various NGs have been implanted in organisms for energy harvesting, sensing, and stimulating nerves and muscles.^[^ ^108^, ^109^, ^110^ ^]^ Two kinds of energy‐harvesting devices are included: PENG and TENG as shown in Figure 6A. Due to the piezoelectric effect, abundant mechanical energy can be harvested by PENG from human bodies including essential motions such as heartbeat, gastrointestinal moving, daily walking and breathing. Since 1880, the piezoelectric effect was discovered by the Curie brothers, many piezoelectric materials were studied. There are several common piezoelectric materials such as zinc oxide (ZnO), lead zirconate titanate (PZT), barium titanate (BaTiO_3_), polyvinyl chloride (PVC), poly (lactic acid) and polyvinylidene fluoride (PVDF). However, inorganic piezoelectric materials such as ZnO and PZT have good performance but are usually rigid and brittle, which causes difficulty to match well with soft tissues. The flexible ZnO nanowire fabricated by Li et al. improved the flexibility of PENG for real‐time rat heart rate monitor, the two‐ends‐bonded piezoelectric nanowire generator can drive the electron motion by mechanical deformation.^[^ ^111^ ^]^ The output voltage and current are usually less than 50 mV and 500 pA, the robustness of the nanowire need to be improved with flexible polymer to isolate it from the biological surrounding. The average output voltage and current from the heartbeat of a live rat were about 3 mV and 30 pA (Figure 6B). While ZnO/rGO can reinforce the mechanical stress with increased local deformation, the enhanced piezoelectric output can be generated. According to Azimi et al., the optimized composite of PVDF nanofibres with ZnO and rGO as hybrid filler generated an output voltage which is nearly tenfold increase than that of the pristine PVDF nanofibres.^[^ ^29^ ^]^ The optimized PENG was sutured on the heart of an adult dog and every heartbeat can generate electrical energy as high as 0.487 μJ which is enough to power commercial pacemakers as demonstrated in Figure 6C. To further improve the output current, Kim et al. fabricated a soft PENG with Mn‐doped PMN‐PZT ((1−*x*)Pb(Mg_1/3_Nb_2/3_)O_3_−(*x*)Pb(Zr, Ti)O_3_) thin film on a PET substrate (Figure 6D). The PMN‐PZT‐based PENG can provide an open‐circuit voltage of 17.8 V and a short‐circuit current of 1.74 μA from porcine rhythmical cardiac contraction and relaxation which is higher than the previous research.^[^ ^112^, ^113^ ^]^ The PENG system successfully enabled ECG signal recording, controlled a light bulb wirelessly and transferred signals. Hypertension (high‐blood pressure) is another health risk factor for heart disease that need long‐term intensive real‐time monitor but traditional air‐filled cuffs for blood pressure test can only provide interrupted results.^[^ ^114^, ^115^ ^]^ To realise real‐time and accurate monitoring of blood pressure, a flexible continuous battery‐free electronic system was developed for in vivo blood pressure monitoring with a miniaturized thin‐film PENG wrapped on the aorta as illustrated in Figure 6E. The devices were encapsulated by the biocompatible flexible polyimide film sandwiched in a 200 μm piezoelectric polarized PVDF thin film and thus can produce current continuously with the periodical expansion and retraction of the aorta. The long‐term stability and possibility of long‐time in vivo use enable the PENG to be a real‐time continuous power source for the visualized blood pressure monitoring system. However, even though PENGs have advantages including high output voltage and good scalability as energy supply, the most widely used material PZT of PENG is toxic and risky to be implanted inside the human body even though it can be sealed by other nontoxic materials such as PDMS and polyimide encapsulation layer, while other biocompatible materials such as ZnO and PVDF can only provide millivolts (mV) and nano‐amperes (nA) output lower than PZT‐based PENG devices.^[^ ^16^ ^]^

Similarly, the TENG relying on the triboelectric effect at the interface between two different dielectrics also provided a promising way to harvest energy from body movements. Generally, the triboelectric effect of TENG occurs between inorganic and organic films with different frictional electric sequences. Various materials in nature such as silk, metal, wood and polymer have such effects. At present, TENG has been successfully applied in biomechanical energy collection for biomedical signal sensing such as respiratory,^[^ ^116^ ^]^ cardiovascular and digestive systems.^[^ ^117^, ^118^ ^]^ For example, Zheng et al. fabricated a self‐powered cardiac pacemaker with an energy supply of TENG harvesting energy from respiration (Figure 6F).^[^ ^30^ ^]^ The size of the TENG device inserted into the left chest skin of a rat is about 1.2 cm wide and 0.2 cm thick. The output voltage and current generated by the TENG device are 3.73 V and 0.14 μA, which was directly applied to power a cardiac pacemaker for heartbeats regulation. Also, the TENG can harvest mechanical energy from gastric peristalsis and can be converted to biphasic electric pulses through contact and separation of the triboelectric layers.^[^ ^119^ ^]^ The generated electric signals were delivered to the fibres of the vagus nerve stimulation (VNS) device and decreased the food intake, achieving the final purpose of weight control as illustrated in Figure 6G.

Comparatively, TENGs have the advantages such as low‐cost, selective materials and high energy conversion efficiency.^[^ ^120^, ^121^ ^]^ However, both TENGs and PENGs as biomechanical energy harvesters face unique challenges such as the stability of continuous energy supply, adaptability to the biological surroundings with different humidity and temperature, long‐term safety inside the body and the limitation for long‐term application.^[^ ^16^ ^]^ Nanogenerators can harvest the energy wirelessly inside living body from periodic motions (including heartbeat and breath) and generate pulsatile power output, but it's unstable and unusable for implantable electronics like cardiovascular electronic devices (e.g. pacemakers, cardioverter defibrillators and cardiac resynchronization therapy devices).^[^ ^110^ ^]^ Appropriate power management is worthy of consideration to boost the application of nanogenerators by rationally designed circuits to convert pulsatile power output into stable power source and increase the energy conversion efficiency. In addition, energy storage devices such as capacitors and batteries are expected to assist the nanogenerators to store the energy as a precaution for temporary energy shortage in IMEs system in future research.

#### 3.2.2 Biofuel cells

**FIGURE 7:**
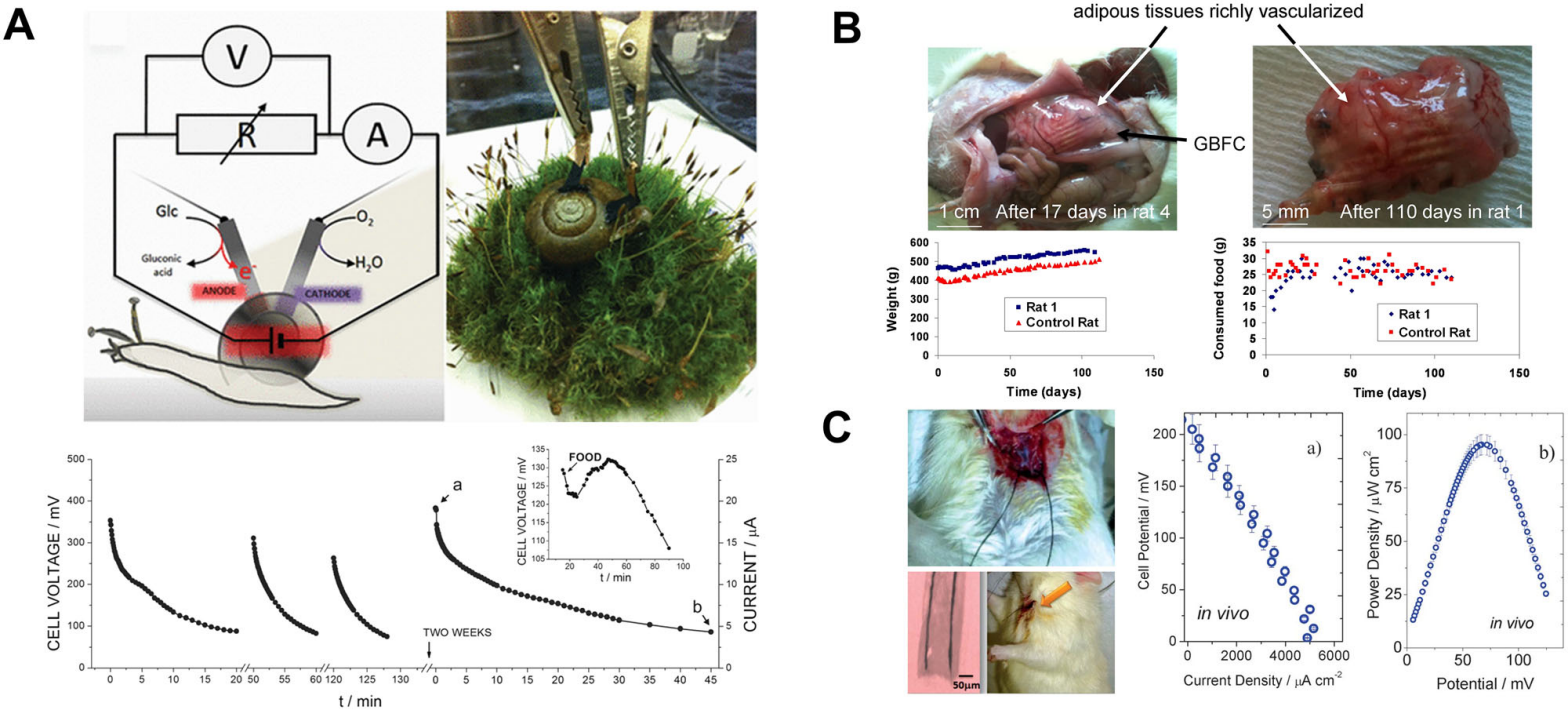
Implantable biofuel cells. (A) The first implanted biofuel cell operating in a living snail and generating electrical power by the consumption of physiologically produced glucose as a fuel. Reproduced with permission.^[^ ^122^ ^]^ Copyright 2012, American Chemical Society. (B) Implanted single glucose biofuel cells in rats as power source devices with no immunological rejection. Reproduced with permission.^[^ ^123^ ^]^ Copyright 2013, Springer Nature. (C) The miniaturized intravenous implantable biofuel cell based on modified flexible carbon fibre electrodes. Reproduced with permission.^[^ ^31^ ^]^ Copyright 2013, Royal Society of Chemistry.

The source ingredients for biofuel cells are abundantly available in our bodies including blood, sweat and tear. Due to the self‐restorative body fluid, the reactant glucose for biofuel cells is abundant enough for long‐term electricity generation. During the oxidation reaction, glucose can generate 12 electrons per molecule and provide energy up to 16 kWh g^−1^.^[^ ^69^ ^]^ By inserting two biofuel electrodes with enzymatic catalysts into the tissue, the biofuel cell can convert chemical energy from glucose oxidization and dioxygen reduction to electricity, and the product gluconolactone will be then absorbed by organisms. It has been reported that biofuel cells have been fabricated and implanted into various organisms (snail, clam, lobster, slug, rat), and the power densities generated by biofuel cells range from 2 to 97 μW.^[^ ^108^ ^]^ Since 2012, sustainable generation of electrical power from a snail was achieved for the first time, and it has been proved that biofuel cells implanted in small animals can operate for several months.^[^ ^122^ ^]^ The reversible decay in the electrical power generation can be observed in Figure 7A, it was reported that the local depletion of glucose at the electrode surface led to the current decay, but the electrical output was restored after feeding the snail followed by slow metabolic processes and glucose diffusion. It is worthy of notice that small species have limited amounts of glucose for biofuel mass transport, which is different from the process in a mammal. To investigate the influence of the implanted glucose biofuel cell on the habits and fitness of the host, researchers investigated the weight of the rat and the food consumption when a biofuel cell based on carbon nanotube/enzyme electrodes was implanted.^[^ ^123^ ^]^ It was implanted into the abdominal cavity and generated an average open‐circuit voltage of 0.57 V and a power output of 38.7 μW, which is enough to power a light‐emitting diode, and as a result, no immunological rejection happens in the rat after the implantation of the biofuel cells for 110 days, as shown in Figure 7B. It shows a promising prospect of the implantation of biofuel cells in a mammal's body fluids for the biomedical electronic power source. To further decrease the dimension of the biofuel cell, a modified flexible carbon fibre microelectrode as intravenous implantable biofuel cell was developed and implanted in the thoracic region of living rats through a catheter.^[^ ^31^ ^]^ An open circuit voltage of approximately 0.125 V was obtained and maximum output power of 95 μW cm^−^^2^ at 80 mV in a living rat was achieved (Figure 7C).

However, the significant limitations of biofuel cells lay in the limited output voltage (less than 1 V) due to the limited oxidation‐reduction potential, which means the biofuel cells can only drive electronics with a low voltage range. There are some improvement methods for the limited output voltage of biofuel cells, such as using a direct current to direct current (DC–DC) converter, charge pumps and connecting several biofuel cells in series.^[^ ^110^, ^124^, ^125^ ^]^ But the DC–DC converter will consume current while connecting several enzymatic electrodes biofuel cells in series will bring more problems such as the expanded volume and implantation difficulty. Also, inflammatory reactions can also occur around the electrode surfaces and phagocytosis of the enzyme coatings of the biofuel cell will shorten the lifespan of the device, so developing a new biocompatible material to protect the electrodes is necessary.^[^ ^31^ ^]^ At the same time, the oxidation reaction of glucose on the enzymatic electrodes will generate by‐products (hydrogen peroxide) which may cause harm to the living body and therefore cause problems during long‐term applications in vivo.^[^ ^108^ ^]^ In this case, the development of new biocompatible materials to protect the enzyme coatings from phagocytosis should be considered for the advancement of this novel long‐term applicable power electronics as a promising sustainable power source for the miniaturized IMEs.

### 3.3 Wireless power transmission technologies

Compared with internal energy harvesting devices, wireless power transmission technology through electromagnetic radiation, acoustic vibrations and optical cells et al. show higher output power up to 500 mW and better stability,^[^ ^126^, ^127^ ^]^ yet the flexibility and dimension are the main challenges for application in IMEs. Four main strategies of external wireless power transmission technologies are included in this section: Far‐field RF radiation, near‐field wireless power transfer (magnetic resonant coupling), photovoltaic power transfer and ultrasonic power transfer.

#### 3.3.1 Far‐field RF radiation

**FIGURE 8:**
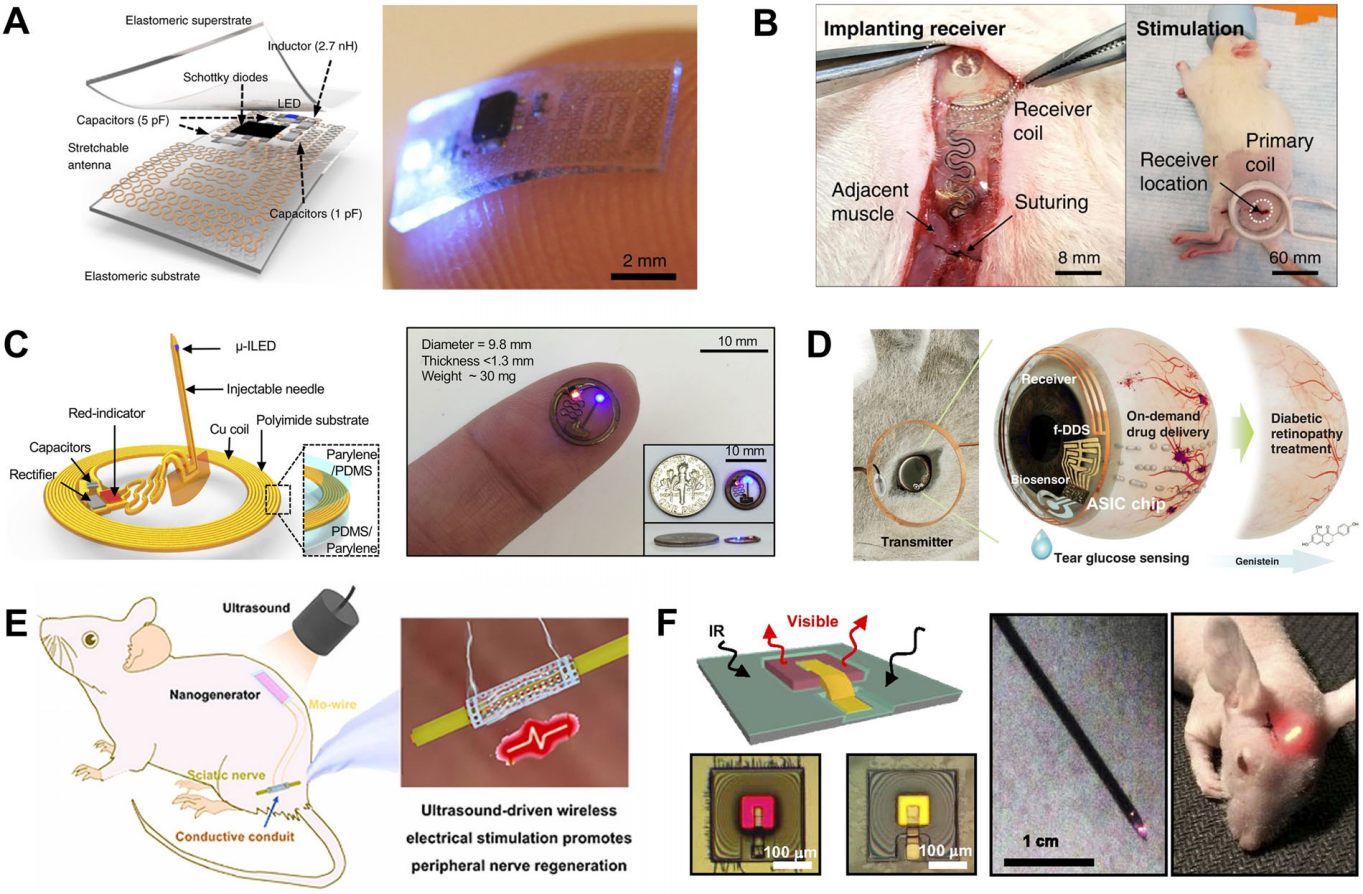
Wireless power transmission technologies for implantable electronics. (A) Miniaturized optoelectronic systems with wireless RF‐powered LEDs for optogenetics. Reproduced with permission.^[^ ^39^ ^]^ Copyright 2015, Springer Nature. (B) Flexible bioresorbable electronic stimulators for neuromuscular regeneration with continuous wireless RF power. Reproduced with permission.^[^ ^24^ ^]^ Copyright 2020, Springer Nature. (C) A thin and flexible optoelectronic implant for optogenetic experiments self‐powered by near‐field wireless power transfer. Reproduced with permission.^[^ ^37^ ^]^ Copyright 2017, Elsevier. (D) Smart contact lens for diabetic diagnosis and therapy with near‐field wireless power transfer and data communication. Reproduced with permission.^[^ ^133^ ^]^ Copyright 2020, Science. (E) Non‐invasive ultrasound‐driven in vivo electrical stimulation for nerve tissue repair monitoring. Reproduced with permission.^[^ ^41^ ^]^ Copyright 2022, Elsevier. (F) Ultraminiaturized wireless implants with infrared‐to‐visible up‐conversion devices as injectable light sources for optogenetic neuromodulation. Reproduced with permission.^[^ ^40^ ^]^ Copyright 2018, Proceedings of the National Academy of Sciences.

Far‐field radio frequency power transfer can be achieved by emitting radio frequency radiation with a frequency ranging from 420 MHz to 2.4 GHz and a wavelength from 0.1 to 1 m, from a transmitting antenna to a receiving antenna, and converting the harvested radio frequency radiation into direct current with a rectifier.^[^ ^128^ ^]^ With specialized primary designs of transmission and receiving antennae, continuous and stable radio frequency power can be delivered over a long distance of up to several meters. With optimized designs of transmission and receiver coil, output voltages larger than 300 mV can be achieved at a distance of 4 mm, and higher voltages can be reached even at 10 V, which can provide enough power for various bioelectronic implants and even soft robots.^[^ ^129^ ^]^ For example, Park et al. introduced a strategy combining flexible neural interfaces with stretchable wireless radio power antenna and rectifying circuits into an integrated miniaturized optoelectronic system to realize the optogenetic modulation of the spinal cord and of peripheral nerves, as illustrated in Figure 8A.^[^ ^39^ ^]^ The antennas with the dimension of 3 mm harvest radio frequency power through capacitive coupling, which enabled the compaction and lightweight with overall weight of ≈16 mg and can accommodate irregular biological interface and natural movements. With a minimally invasive and flexible structure, it can be expected to be used in chronic studies. Figure 8B shows a soft wireless bioresorbable neuromuscular stimulation platform for nerve regeneration with a radio frequency wireless receiver antenna as the power source.^[^ ^24^ ^]^ The stimulator encapsulated by bioresorbable polyurethane can work for more than one month with good stability. According to the new Institute of Electrical and Electronic Engineers (IEEE) standard on human exposure to radio frequency radiation, the safe range of frequency is from 3 kHz to 100 GHz.^[^ ^130^ ^]^ Based on the absorption rate of tissue in most parts of the human body, the maximum exposure of radio frequency radiation amount is about 2 W kg^−1^, and incident power densities range from 1000 W m^−2^ at 100 kHz to 10 W m^−^^2^ at 100 GHz with the minimum value of 2 W m^−^^2^ between 30 and 400 MHz, which is established to protect the body from damage caused by tissue heating. Also, the transmission efficiency will be influenced by the orientations of the transmitting antenna and receiving antenna, and the surrounding obstacles such as metal and human tissue containing moisture will lead to heat generation and limited operating ranges from 0.1 to 3 m.^[^ ^131^ ^]^ To avoid these disadvantages, a specialised design is urgently needed for the implantable antenna. For IMEs using wireless far‐field radio frequency power transfer as energy supply method, long‐term applications should be considered carefully due to the safety issues that radio frequency radiation exposure would bring to living organisms.

#### 3.3.2 Near‐field wireless power transfer

The near‐field wireless power transfer method adopts magnetic resonant coupling (non‐radiative electromagnetic energy) and relies on inductive coupling between a transmitting coil and a receiving coil.^[^ ^16^ ^]^ This strategy can transmit wireless power with high efficiency over short distances of about several centimetres. Due to the non‐radiative nature of magnetic resonant coupling with frequencies ranging from 100 kHz to 200 MHz, the near‐field wireless power transfer method is relatively insensitive to changes in dielectric environments such as moisture in tissues and to the presence of obstacles such as metals compared with far‐field radio frequency power transfer. Also, near‐field wireless power transfer working at a low working frequency applied in IMEs will generate less heat and reduce tissue absorption and therefore can be applied in complex biological surroundings, which minimizes safety concerns during long‐term operation. Meanwhile, data transmission or remote wireless control can also be realised when using wireless near‐field power transfer since near‐field communication (NFC) protocols can be used at the same carrier frequency.^[^ ^132^ ^]^ With near‐field wireless power transfer, a fully implantable subcutaneous electronic system with an ultrathin probe and a micro light emitting diode for optogenetic stimulation of regions in the deep brain can be wirelessly powered with reliable operation and good chronic stability, as illustrated in Figure 8C.^[^ ^37^ ^]^ Furthermore, the near‐field wireless power transfer and NFC are also applied in the biomedical smart contact lens device with modules including ultrathin electrical circuits, microcontroller chip for real‐time electrochemical biosensing and functions including on‐demand controlled drug delivery, the near‐field wireless power transfer and data communication (Figure 8D).^[^ ^133^ ^]^ The smart contact lens system is demonstrated to be feasible for application in non‐invasive and continuous diabetic diagnosis and diabetic retinopathy therapy. It is reported that near‐field wireless power via magnetic resonant coupling is a reliable energy supply method with power up to 12 mW for small rodent‐sized devices and about 13 mW cm^−^^2^ can be achieved.^[^ ^134^ ^]^ With these advantages including low working frequency, less produced heat, minimized safety concerns, insensitivity to environments and data communication functions, near‐field wireless power transfer method significantly promoted the development of wireless IMEs for various applications (e.g. localized tissue oximetry, bioresorbable monitoring, optogenetic stimulation, pharmacological modulation). In comparison, this energy supply strategy is superior to in vivo energy‐harvesting strategies in terms of the amount and stability of output power.

#### 3.3.3 Ultrasonic power transfer

The ultrasonic power transfer wireless energy harvesting method is an emerging energy transfer technology by converting ultrasound waves to electricity through piezoelectric semiconducting coupling.^[^ ^135^ ^]^ Ultrasound is an acoustic sound wave with a frequency higher than 20 kHz but much shorter wavelengths than radio frequency radiation, energy transmission to IMEs can be achieved with high spatial resolution.^[^ ^136^ ^]^ Therefore, compared with traditional wireless energy harvesting based on electromagnetic coupling, ultrasonic power transfer possesses several advantages in the applications of IMEs, such as small attenuation of ultrasonic power in biological tissues and excellent spatial resolution. For example, a biodegradable PENG driven by external ultrasound power without any transcutaneous leads was fabricated for sustained delivery of in vivo electrical stimulation to promote the repair of peripheral nerve injuries.^[^ ^41^ ^]^ Under an ultrasound power intensity of 0.7 W cm^−^^2^ for short‐term excitation, the current for stimulation driven by ultrasonic power is about 40 μA and the voltage of single stimulation is 10 mV with the pulse width of 1 ms demonstrating the feasibility of non‐invasive monitoring of the nerve repairing dynamics (Figure 8E). However, this feature also brings disadvantages, for example, the ultrasonic waves propagate in a specific direction, and the coupling efficiency highly rely on the good alignment and orientations between the external transducer and the implanted receiver.^[^ ^35^ ^]^ To avoid a substantial impedance mismatch at the tissue‐air interface, it is essential to make sure of close contact of the transducer with the skin. Though ultrasound could be absorbed in a relatively moderate degree by soft tissues and is possible to realize large penetration depths,^[^ ^137^ ^]^ but in the meantime it may also induce nonnegligible heating effects in highly absorbing bone area,^[^ ^138^ ^]^ and cavitation may also occur at low ultrasonic frequencies which may bring safety problems.^[^ ^139^ ^]^ Also, ultrasonic power transfer devices are usually rigid and large in size, which is not suitable for curvilinear and soft tissue and organs. Recent advances in ultrasonic power sources with sufficient penetration depth and excellent spatial resolution represented a promising application in non‐invasive power sources for IMEs and challenges remain to be solved regarding the flexibility and the coordination with drag force generated in harsh environments in human body.

#### 3.3.4 Photovoltaic power transfer

Photovoltaic power transfer captures the energy from electromagnetic radiation in the form of visible and near‐infrared light and converts them into electric energy by photodiodes.^[^ ^140^ ^]^ For IMEs, photovoltaic power transfer devices need to be enfolded in soft living tissues, such as skin, fats and muscles, so within the near‐infrared spectrum (700–2500 nm), high‐scattering tissue can be penetrated more efficiently by the light. For optogenetic experiments, optical fibres were required to be inserted into the brain tissue with another end attached to a remotely located light source such as a laser or light‐emitting diode, but the fibre tethers limited the movement of the animals and caused entanglement which led to the prohibition of neuroscience studies regarding the social interactions and home cage manipulations of small animals in complex environments.^[^ ^141^ ^]^ Compared to previously reported systems, injectable optoelectronics under cellular‐scale for wireless optogenetics has been reported with six times smaller dimensions and thirty‐fold lighter weight.^[^ ^51^ ^]^ In this case, wireless, ultra‐miniaturized LEDs that can be implanted directly into the brain will well solve the constraints, the photovoltaic cells can provide wireless power for the optogenetic electronics and can significantly expand the movement range of the experimental animals. The enhanced photovoltaic systems with dual junction gallium arsenide (GaAs) solar cells array have an open‐circuit voltage of 2.31 V and short‐circuit currents of 11.17 mA with the efficiency of 25%, and can provide 4 V to support the wireless power for control logic circuits and optogenetic stimulation under illumination with sunlight and desk lamp.^[^ ^141^ ^]^ It can supply the power for blue and yellow micro light emitting diodes at intensities of 3.5 and 2.3 mW mm^−^^2^. Furthermore, optoelectronic devices based on semiconductor optoelectronic technologies have been developed for power harvesting in ultraminiaturized wireless implants for optogenetic neuromodulation.^[^ ^40^ ^]^ A thin‐film optoelectronic device combining an infrared double‐junction photodetector and a visible LED was designed and realised near‐infrared (≈810 nm) to visible (630 nm (red) or 590 nm (yellow)) up conversion (Figure 8F). It can capture photons from external incoherent low‐power infrared LED bulbs and generates an upconverted red emission power density of about 1.1 mW mm^−^^2^ on the device surface, which can effectively manipulate the activated ChrimsonR‐expressing neurons.^[^ ^142^ ^]^ As an implantable photovoltaic power source for wireless optogenetic control of in vitro and in vivo neuroactivities, it can be used for deep‐tissue light stimulation and provided unprecedented potential for the application in optical bio interface. Compared with systems with far‐field RF power, the photovoltaic power transfer can achieve high conversion efficiency with solar cells (10 times less power required compared with far‐field power).^[^ ^141^ ^]^ However, similar to all the external power transfer methods, the output power of solar cells strongly depends on incident angles,^[^ ^143^ ^]^ additional optical imaging or tracking systems are necessary for the optics focusing, which will put a limitation on the experimental area and lead to constrained behaviours of experimental animals and human body using this power strategy for IMEs.

## 4 CONCLUSIONS AND PERSPECTIVES

The past decades have witnessed revolutionary changes in the field of IMEs owing to the surging demand not only for the functional electrical therapy of chronic degenerative diseases but also bio‐signals for health care monitoring with high fidelity and stability. The rapid development of miniaturized implantable electronics in recent years reveals the urgent demand for minimally invasive power sources. Traditional bulky and rigid power devices including primary batteries packaged in metal cases can no longer satisfy the requirements of the state‐of‐the‐art implantable electronic system regarding flexibility, biocompatibility, durability, lightweight and minimal invasion. As an overall conclusion of the recent advances in miniaturized IMEs and power strategies for the system, this review provides a comprehensive summary of the historical development of implantable electronics and the applicable alternative miniaturized power sources for the advanced miniaturized IMEs system with an outlook for challenges in the future development. From the milestones in the development history of implantable electronics, the tendency towards minimizing the incision size and optimizing biocompatibility is obvious. With the facilitation of the recent advanced technologies in microfabrication technologies and biocompatible materials, IMEs system will be developed towards non‐invasive, ultra‐flexible, bioresorbable, wireless and multifunctional to therefore achieve painless implantation and high‐accuracy bio‐functional monitoring.

To discuss the applicable minimally invasive power sources with different mechanisms for various IMEs, three kinds of power sources including energy storage devices, human body energy harvesters and wireless power transfer were summarized: (i) For the stable energy storage devices, the biodegradability feature enables single‐used primary batteries to serve as a short‐term stable power source for transient bioelectronics with no need for surgical removal thanks to the fully degradable and biocompatible materials, whereas the exploration of controllable packaging methods and clear dissolution mechanisms still need further study. (ii) Rechargeable batteries and supercapacitors with 1D fibre configuration are desirable for an injectable bioelectronic system requiring sustainable long‐term power sources due to their good stability and rechargeability. (iii) In addition, human body energy harvesters including PENG, TENG and biofuel cells as a permanent energy source can be implanted once for all and support the IMEs to finish the missions during the lifetime of the hosts, however, the stability of continuous power supply and long‐term safety inside the body represents the main limitation for their long‐term application. (iv) Finally, wireless power transfer including near‐field magnetic resonant coupling, far‐field RF radiation, PV power transfer and ultrasonic power transfer can provide higher output power as a direct power source or assistant external power source to charge the energy storage devices, through electromagnetic and acoustic waves, wireless power can be transferred to avoid the limitations caused by tethers, but concerns of safety issues brought by the exposure limit of the human body need further consideration.

It is still challenging to realize the self‐powered minimally invasive IMEs with long‐term stable functions through a service time reaching or exceeding the human lifespan. The complex and integrated independent system with power source management, biomedical functions and wireless communication operating as a whole in the human body should be further explored. In such kind of advanced electronic system, developments of power source should be considered through the following aspects.

1. Structure and compatibility optimization. Advanced nano fabrication and micro‐dimension packaging technologies should be introduced to promote miniaturization, high integration, and flexibility and minimize the disturbance/harm to biological functions. Multiphysics simulations can be more broadly employed to optimize device structure and simulate biochemical interaction between device and biological environment.
2. Power management optimization. Pulsatile or unstable energy harvesters (e.g. nanogenerators and biofuel cells) with well‐designed electrical circuits should be combined to provide stable and sustainable power.
3. Wireless communication and data transfer. Communication networks with micro antenna and transmitters can be further developed to provide real‐time, remote and non‐constructive data transfer.
4. Multifunction integration. With improved power performance, multifunction electronic modules (sensors, etc.) can be integrated and powered for multimodal monitoring of various bio‐electrochemical signals.
5. Feasibility evaluation. More specified application and testing in different biological environments (such as biofluid with moisture, corrosive gastric acid, narrow blood vessel, cardiac surface with dramatic stretching and shrinking) should be conducted to evaluate the tolerance toward harsh environment and long‐term operation stability.
6. Characterization and degradation analysis. Standard evaluation (such as energy output, energy conversion efficiency, etc.) should be established for the overall performance customization and optimization. Multiscale/dimensional operando characterizations should be introduced to investigate interfacial interactions between implanted self‐powered IMEs and the biological surroundings to understand the degradation mechanism.

With the advancement in these frontiers, it is promising to achieve miniaturization and multifunctional combination of minimally invasive power sources driven IMEs system to realize pain‐free health monitor and biomedical treatment with high accuracy and fidelity in the near future.

## CONFLICT OF INTEREST STATEMENT

The authors declare no conflicts of interest.
